# Revealing the CO Coverage-Driven C–C Coupling Mechanism for Electrochemical CO_2_ Reduction on Cu_2_O Nanocubes *via Operando* Raman Spectroscopy

**DOI:** 10.1021/acscatal.1c01478

**Published:** 2021-06-11

**Authors:** Chao Zhan, Federico Dattila, Clara Rettenmaier, Arno Bergmann, Stefanie Kühl, Rodrigo García-Muelas, Núria López, Beatriz Roldan Cuenya

**Affiliations:** † Department of Interface Science, Fritz-Haber Institute of the Max-Planck Society, Faradayweg 4-6, 14195 Berlin, Germany; ‡ Institute of Chemical Research of Catalonia (ICIQ), The Barcelona Institute of Science and Technology (BIST), Av. Països Catalans 16, 43007 Tarragona, Spain

**Keywords:** operando Raman spectroscopy, CO_2_ reduction, Cu_2_O nanocubes, C-C coupling

## Abstract

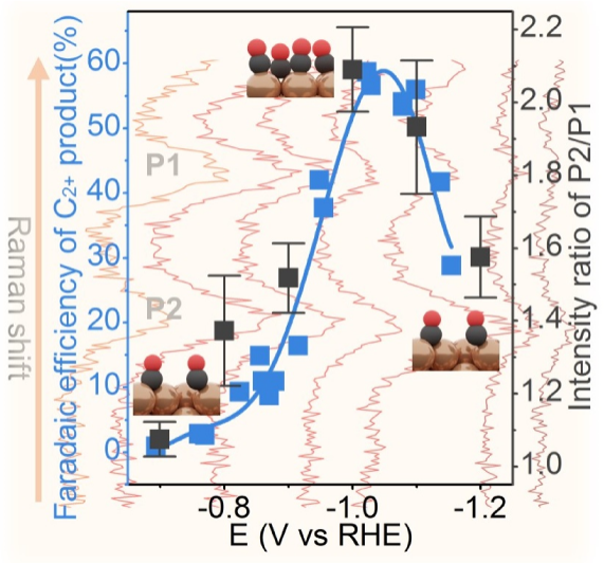

Electrochemical reduction of carbon dioxide (CO_2_RR) is an attractive route to close the carbon cycle and potentially turn CO_2_ into valuable chemicals and fuels. However, the highly selective generation of multicarbon products remains a challenge, suffering from poor mechanistic understanding. Herein, we used *operando* Raman spectroscopy to track the potential-dependent reduction of Cu_2_O nanocubes and the surface coverage of reaction intermediates. In particular, we discovered that the potential-dependent intensity ratio of the Cu–CO stretching band to the CO rotation band follows a volcano trend similar to the CO_2_RR Faradaic efficiency for multicarbon products. By combining *operando* spectroscopic insights with Density Functional Theory, we proved that this ratio is determined by the CO coverage and that a direct correlation exists between the potential-dependent CO coverage, the preferred C–C coupling configuration, and the selectivity to C_2+_ products. Thus, *operando* Raman spectroscopy can serve as an effective method to quantify the coverage of surface intermediates during an electrocatalytic reaction.

## Introduction

The electrochemical reduction of carbon dioxide (CO_2_RR), powered by renewable electricity, is an attractive route to convert CO_2_ into valuable products, thereby closing the anthropogenic carbon cycle and transforming intermittent energy into chemical energy to provide fuels and feedstocks.^1^ Although numerous efforts have been made, the highly effective and selective generation of economically desirable products remains a great challenge, especially for multicarbon chemicals (C_2+_) such as ethylene and ethanol with higher energy density and wider applicability.^2−6^ Concurrently, processes involving C–C bond formation are of great interest and significance to fundamental research.

Cu-based materials are known to be the most active catalysts for CO_2_RR to yield C_2+_ products in significant amounts.^2,7^ Thus, many experimental and theoretical studies have focused on understanding the C–C coupling mechanism on Cu surfaces.^8−12^ From a modeling perspective, CO is considered as one of the key intermediates in CO_2_RR since it can dimerize to form OCCO species or be hydrogenated to form CHO species.^9,13,51^ Experimentally, it is known that the onset potential for the formation of C_2_H_4_ starts 300–400 mV more negative than the onset potential for CO evolution and that the Faradaic efficiency (F.E.) of the C_2+_ products shows a volcano dependence on the applied potential.^7,14,15^ Specific CO adsorption configurations are considered to be crucial for OC–CO dimerization,^4,16^ and a link between the CO coverage and the formation of C_2+_ products has been proposed.^17,18^ However, key experimental observations remained absent, and it is still a great challenge to quantify the CO coverage at the solid–liquid interface during CO_2_RR and give an unambiguous explanation on the potential-dependent C_2+_ F.E. at the molecular scale. Furthermore, a full mechanistic understanding of the C–C coupling at certain given CO coverages and adsorption configurations is still not conclusive. Therefore, an *operando* method is highly desirable to determine the surface coverage of CO during CO_2_RR.

Surface-enhanced Raman spectroscopy (SERS), detecting vibrational and rotational information in a broad spectral range with high surface sensitivity, allows to investigate the electrochemical solid–liquid interface and the interaction of surface intermediates with the active electrode.^19−21^ Nonetheless, the widespread application of this method is limited by the necessity of using plasmonic materials. Fortunately, nanostructured Cu materials display typical plasmonic effects that can enhance the Raman signals of surface species and improve the detection limits; thus, more attention has been paid to the use of Raman to investigate the CO adsorption and configuration during the CO_2_RR process.^22−25^ However, since the surface enhancement effect is highly related to the local nanostructure, the quantification of adsorbate surface concentrations directly from the spectral Raman intensity is hindered.

Herein, we used *operando* Raman spectroscopy combined with quasi-*in situ* Cu LMM X-ray Auger electron spectroscopy (XAES) to reveal the transformations of the electrode–liquid interface during CO_2_RR over Cu_2_O nanocube electrocatalysts. In particular, we discovered that the intensity ratio of the Cu–CO stretching to the CO rotation band is determined by the CO coverage. This observation was confirmed and explained by *operando* Raman experiments in CO-rich electrolytes with different CO concentrations and theoretical investigations based on Density Functional Theory (DFT). We further established a direct correlation between the C_2+_ product selectivity, the potential-dependent CO surface coverage, and CO adsorption configurations under reduction conditions. Our work demonstrates that *operando* SERS combined with DFT is an integrated methodology for investigating the electrochemical solid–liquid interface and quantifying the coverage of surface intermediates during electrocatalytic reactions.

## Results and Discussion

**Figure 1:**
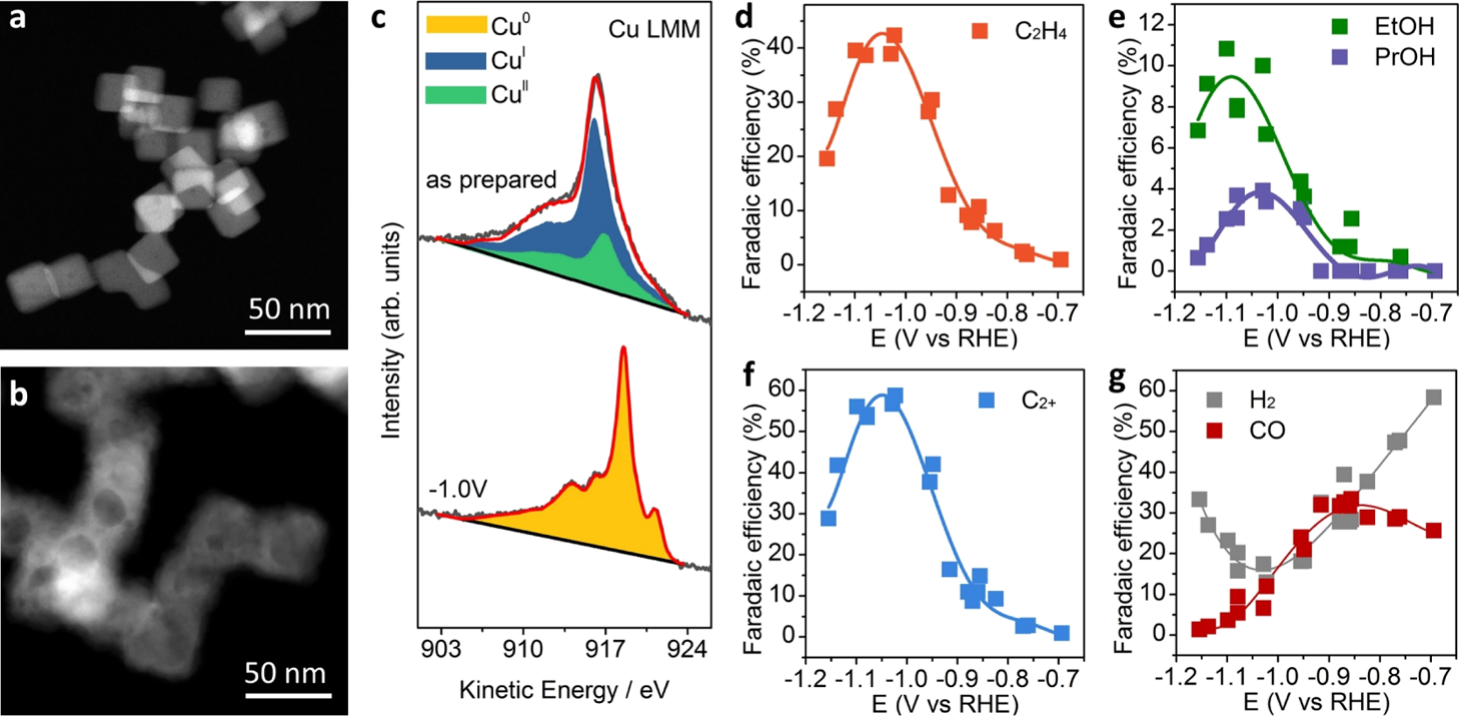
Structural and chemical characterization as well as CO_2_RR performance of Cu_2_O nanocubes. TEM images of Cu_2_O nanocubes in their as-prepared state (a) and after 1 h CO_2_RR at −1.0 V_RHE_ (b). (c) Quasi-*in situ* Cu LMM XAES spectra of Cu_2_O nanocubes in the as-prepared state and after 1 h of CO_2_RR at −1.0 V_RHE_ without air exposure. Potential-dependent F.E. of (d) ethylene, (e) ethanol (EtOH) and 1-propanol (PrOH), (f) the sum of all C_2+_ products, and (g) H_2_ and CO obtained after 1 h of CO_2_RR. Solid lines are guides for the eye. All electrochemical experiments were conducted in 0.1 M KHCO_3_, and the electrode potentials are given *vs* the RHE.

Well-defined and surfactant-free ∼25 nm Cu_2_O nanocubes were used as a model system (Figures 1a, S1, S2). The X-ray diffraction pattern confirms the sole presence of Cu_2_O, and Rietveld refinement reveals a structural coherence length of ∼29 nm and a lattice parameter, *a*, of 4.267(2) Å (Figure S3). Linear combination analysis of quasi-*in situ* Cu LMM XAES data of the as-prepared nanocubes (Figure 1c) indicates a surface composition of about 80% Cu(I) and 20% Cu(II) species. After 1 h of CO_2_RR at −1.0 V_RHE_ in 0.1 M KHCO_3_, the cubic morphology was partially retained, although hollow Cu structures were observed (Figures 1b, S4, S5), consistent with previous reports.^26^ It should be noted that the slight differences in the cube size observed in the transmission electron microscopy (TEM) images presented likely originate from the size distribution already present in the as-prepared cubes. A further plausible explanation is the redeposition of small Cu particles from dissolved Cu species in the electrolyte originating from the hollowed cubes that were observed during the CO_2_RR process. The surface of the Cu_2_O nanocubes was fully reduced to metallic Cu after CO_2_RR (Figure 1c), in agreement with the Cu 2p X-ray photoelectron spectroscopy data (Figure S6). For these experiments, the sample was transferred under an inert atmosphere between the electrochemical cell and the directly interfaced XAES ultrahigh vacuum analysis chamber (Figure S7).

Figure 1d–g shows the F.E.s of CO_2_RR products which vary strongly with the applied potential (Figures S8 and S9). A typical volcano dependence of the F.E. on the applied potential appears for the C_2+_ products, which reaches a maximum of 60% at about −1.05 V_RHE_. The CO F.E. decreases with the potential from about −0.85 to −1.2 V_RHE_. The potential-dependent F.E. of H_2_ opposes the trend obtained for the C_2+_ products, with a minimum value at around −1.0 V_RHE_. The CH_4_ F.E. increases with the potential from about −1.0 to −1.2 V_RHE_. Similar potential-dependent F.E.s of CO_2_RR products have been widely reported and discussed experimentally and theoretically,^7,14,15,27^ but a molecular understanding is still lacking.

**Figure 2:**
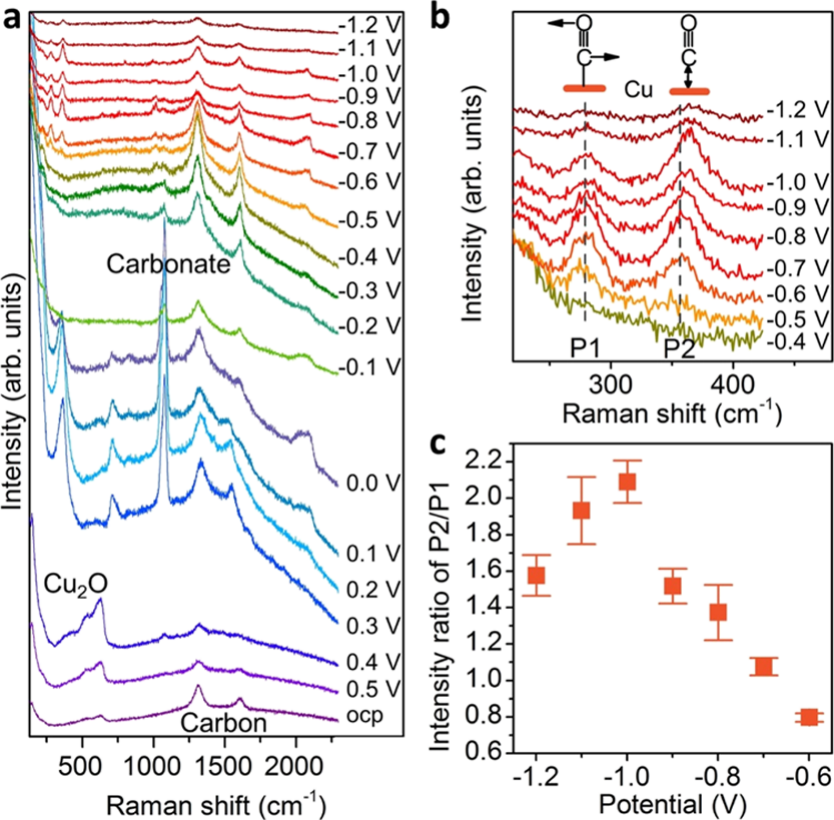
*Operando* Raman spectroscopy data on Cu_2_O nanocubes during CO_2_RR. (a) Raman spectra of Cu_2_O nanocubes acquired during CO_2_RR for potentials ranging from the OCP to −1.2 V_RHE_. (b) Zoom-in spectra of the restricted rotation of adsorbed CO (P1) and Cu–CO stretching (P2) from −0.4 to −1.2 V_RHE_. (c) Potential-dependent intensity ratio of P2 to P1. The error bars were obtained as standard deviation (see the Supporting Information for details). All electrochemical experiments were conducted in 0.1 M KHCO_3_, and the electrode potentials are given *vs* the RHE.

*Operando* SERS measurements were carried out to investigate the catalyst structure and surface adsorbates during CO_2_RR as well as their dynamics (see the experimental setup in Figure S10). Figure 2a displays the *operando* Raman spectra acquired on the same position of a glassy carbon electrode decorated with Cu_2_O nanocubes as a function of the applied potential in a CO_2_-saturated 0.1 M KHCO_3_ electrolyte. The glassy carbon substrate exhibits Raman peaks at 1313 and 1616 cm^–1^ for potentials ranging from the open circuit potential (OCP) to −1.2 V_RHE_ (Figure S11). The potential-dependent change of these two peaks in Figure 2a is mainly due to the transformation of Cu_2_O to metallic Cu and the formation of bubbles on the electrode surface during CO_2_RR. Consecutive Raman spectra at OCP prove the stability of the electrode under the measurement conditions employed (Figure S12).

Raman peaks at 415, 530, and 625 cm^–1^ belong to Cu_2_O.^28,29^ When the potential decreases to +0.3 V_RHE_, these peaks disappear, and a new peak at about 360 cm^–1^ evolves (Figure S13). The Raman shift of this band is similar to that of the Cu–CO stretching of adsorbed CO, but its assignment is still under debate.^24,30,31^ Our data shows that this band shifts to lower wavenumbers as the potential decreases (Figure S14), just opposite to the change of the Cu–CO stretching band (Figure 2b). The red shift of the wavenumber and the relative high onset potential preliminarily rule out its assignment to the Cu–CO stretching band. Interestingly, the 360 cm^–1^ peak is accompanied by another peak at 706 cm^–1^ from +0.3 to 0.0 V_RHE_, which has been assigned to surface hydroxyl species.^31,32^ Concurrently, a strong signal of carbonate species at 1077 cm^–1^ is detected, in line with previous experimental studies and DFT benchmarks (Table S1).^24,30,52,53^ In order to provide a reasonable assignment, systematic control experiments were carried out. During the backward scan from CO_2_RR conditions to the OCP (Figures S15 and S16), with only metallic Cu, no peaks at such wavenumber were observed in the similar potential range, which means that this peak is not related to reaction intermediates of CO_2_RR but related to the reduction of copper oxide species. Control experiments in Ar-saturated KHCO_3_ showed the same peaks at a similar potential (Figure S17), indicating that this band is independent of the CO_2_ electrolyte saturation. Without carbonate ions in the electrolyte, these bands disappeared as seen in Ar-saturated NaClO_4_ (Figure S18). Thus, we assign this band at 360 cm^–1^ to the surface copper carbonate species formed during the reduction of copper oxide species in the presence of carbonate electrolyte ions (KHCO_3_) and hydroxyl species,^33^ in agreement with Raman spectra of malachite and azurite,^34^ and DFT vibrational frequencies of carbonate on Cu (Table S1).

At about −0.1 V_RHE_, the electrode with the surface copper carbonate species is reduced to metallic Cu, consistent with the cyclic voltammogram (Figure S19). Furthermore, during the reduction process, no significant CO signal is detected. A peak around 2000 cm^–1^ is observed; however, this feature disappears at about −0.4 V_RHE_. The same vibrational fingerprint is detected at a similar potential in a control experiment using Ar-saturated KHCO_3_ as electrolyte (Figure S17). In the literature, this peak was previously attributed to H adsorbed on Cu.^35^ From −0.5 V_RHE_, the presence of adsorbed CO is demonstrated by the Raman peaks located at 280, 355–370, and 1970–2110 cm^–1^, corresponding to the restricted rotation of adsorbed CO (P1), Cu–CO stretching (P2), and C–O stretching, respectively (Figure 2a,b).^36−38^ In some reports, the broad C–O stretching band was attributed to different CO adsorption configurations or sites, including bridge-bonded and atop-bonded CO or CO adsorbed on terrace and defect sites.^4,53,39^ Moreover, the P1 and P2 bands, which reflect the interaction between reaction intermediates and the Cu electrode surface, change regularly with the potential. P2 displays a blue shift in the peak frequencies as the electrode potential decreases (Figure 2b). A similar potential-dependent phenomenon has been reported for carbon monoxide chemisorbed on a platinum surface, and it was attributed to the electrochemical Stark effect.^40^ Because the influence of dipole–dipole coupling interactions on the Pt–CO vibration has been proven negligible, the Raman shift of the Pt–CO band can be considered an indicator for potential-induced changes in the bonding strength and bond length of Pt–CO.^40^ The same approach might be applied to our system, since Cu exhibits lower binding strength to CO as compared to Pt, and therefore, it is plausible that the blue shift of the Cu–CO vibration frequencies observed reflect a stronger Cu–CO bond at more negative potentials. At −0.5 V_RHE_, the spectral intensity of P1 is significantly stronger than that of P2, and as the potential shifts negatively, P2 becomes gradually stronger than P1. Figure 2c displays the potential dependence of the intensity ratio of P2 and P1, which exhibits a volcano-type profile increasing from P2/P1 values of 0.7 to 1.4 as the potential decreases from −0.6 to −0.9 V_RHE_ and reaching a maximum at −1.0 V_RHE_ and then decreasing quickly (Figures S20 and S21).

Most importantly, the intensity ratio of these two peaks as a function of the applied potential follows a similar trend as the CO_2_RR F.E. of the C_2+_ products. Through carefully reviewing and reanalyzing the Raman data in previous literature, we found that higher P2/P1 Raman peak ratios were associated with higher F.E.s for C_2+_ products in a variety of different catalysts, for example, CuAg nanowires *versus* Cu nanowires.^22^ Previous studies conducted with synchrotron radiation in the far-infrared range suggested that the intensities of these two peaks could be related to the surface coverage of CO on Cu in vacuum, although the Fano-like infrared peak made it difficult to precisely extract the ratio.^41^ Thus, a reasonable assumption is that our data reflect the CO coverage at the solid–liquid interface during the CO_2_RR process. Such a ratio may also reveal information on the electrochemical double layer, including the local pH, coadsorption, or solution environment.

**Figure 3:**
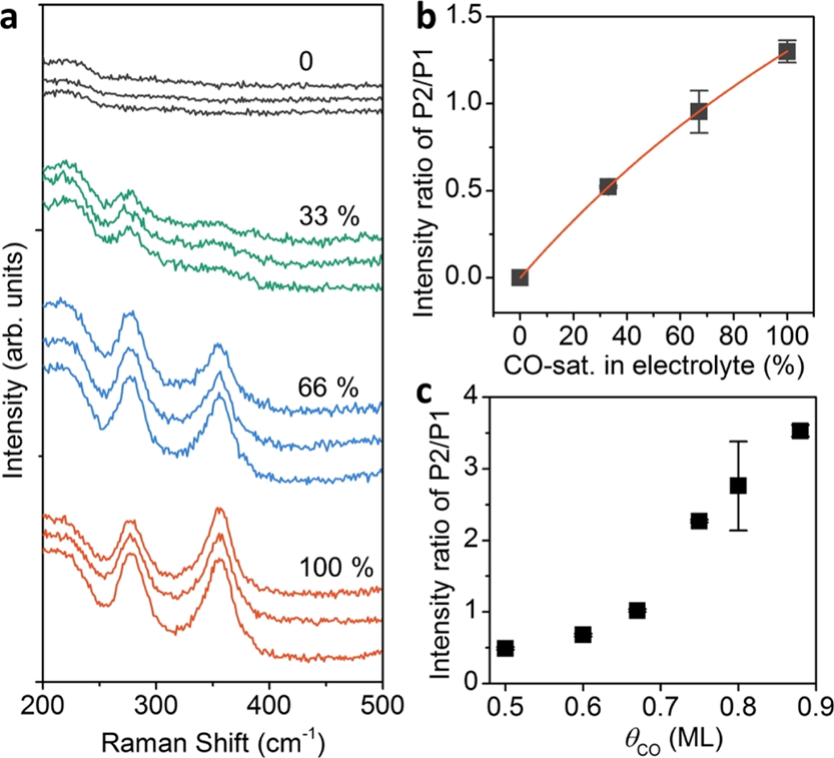
Raman spectra of adsorbed CO on Cu with different CO concentrations in the 0.1 M KHCO_3_ electrolyte. (a) *Operando* Raman spectra of Cu_2_O nanocubes in 0.1 M KHCO_3_ electrolyte with different CO concentrations at −0.52 V_RHE_. We mixed the CO-saturated KHCO_3_ with the Ar-saturated KHCO_3_ to prepare the CO-rich KHCO_3_ with different CO concentrations. The percentage represents the volume fraction of CO-saturated KHCO_3_ in the electrolyte from 0 to 100%. The experiments with different CO concentrations are repeated three times. (b) Intensity ratio of the P2/P1 Raman peaks as a function of the CO concentration. The red line shows the fitting result based on a Langmuir equation. (c) Theoretical benchmark of the P2/P1 intensity ratio *vs* CO surface coverage, θ_CO_ (Tables S4–S6).

To gain further insight into the evolution of the P2/P1 Raman peak ratio, we performed *operando* measurements in the presence of CO as well as DFT vibrational analysis for different CO coverages on the Cu(100) surface (Figure 3. First, we determined the optimal electrode potential to follow the CO adsorption on Cu in the CO-saturated KHCO_3_ electrolyte. We identified a potential window between about −0.32 V_RHE_ and −0.62 V_RHE_ in which adsorbed CO can be detected and the P2/P1 peak ratio also increases with decreasing potential (Figure S22). The CO bands disappeared at −0.72 V_RHE_, indicating the electrochemical conversion of CO. We also verified the stability of CO at different potentials in time-dependent CORR Raman data, with the P2/P1 intensity peak ratio decreasing due to the consumption of CO at −0.72 V_RHE_ and below (Figure S23). To minimize the conversion of CO, we carried out *operando* Raman measurements at −0.52 V_RHE_ in electrolytes with different CO concentrations (Figures S24 and S25). The volume fraction of CO-saturated KHCO_3_ in the electrolyte is used to describe the CO concentration. In principle, the CO surface coverage can be adjusted by controlling the CO concentration in the electrolyte, and their relationship is usually described by the Langmuir’s equation. As shown in Figure 3a, the P2/P1 intensity ratio increases with increasing CO concentration, following Langmuir equation (Figure 3b). Similar results were observed in the CO-containing NaClO_4_ solution (Figures S26–S30). These data demonstrate that the P2/P1 Raman peak ratio is a valid measure of the surface coverage of CO.

We then performed DFT simulations of CO vibrational frequencies on Cu(100) for different CO surface coverages and adsorption configurations (0.11–0.88 ML) to link the experimental Raman spectral features to the relevant CO surface coverage (see Computational Details in the Supporting Information). Cu(100) terraces have been reported on OD-Cu under reduction conditions,^6^ and this facet is suggested to be the most active toward ethylene production.^42^ At relatively high surface coverages, θ_CO_ ≥ 0.6 ML, CO adsorbs in a mix of atop and bridge configurations,^16,43^ an experimental observation which is correctly reproduced by our DFT results (Table S2). To prove the robustness of our computational setup, we benchmarked the CO binding energy as a function of Hubbard’s *U*_eff_,^44^ Cu slab thicknesses, and applied electric field (Figures S31 and S32, Tables S3). A four-layer Cu(100) model without Hubbard correction on C(O) 2p orbitals provided excellent agreement with the experimental results for CO binding energy for coverages expected under CO_2_ reduction conditions, θ_CO_ ≥ 0.5 ML (Table S2).^43,44^

Figure 3c shows the calculated P2/P1 peak ratio as a function of the CO coverage for θ_CO_ ≥ 0.5 ML (Tables S4–S6) which we extracted from DFT-derived Raman spectra (Figure S33). A clear increase of this ratio is observed with increasing CO coverage, and the values obtained theoretically are comparable with those experimentally determined. The increase of the P2/P1 ratio is correlated with a larger population of weakly bound CO_atop_ at high surface coverage, for which C=O rotation (P1) is increasingly reduced. Furthermore, by comparing the theoretical (Figure 3c) and experimental data (Figure 2c), we can associate the intensity ratio detected experimentally under CO_2_RR conditions at −0.6 and −1.0 V_RHE_ (P2/P1 = 0.8 and 2.1) with a DFT CO surface coverage of 0.60 to 0.75 monolayer (ML), respectively (Table S6). Thus, we confirmed that the intensity ratio of the frustrated Cu–CO rotation and the Cu–CO stretching is not only a valid measure of the CO coverage but also linked to a predominant CO binding motif to the Cu surface.

**Figure 4:**
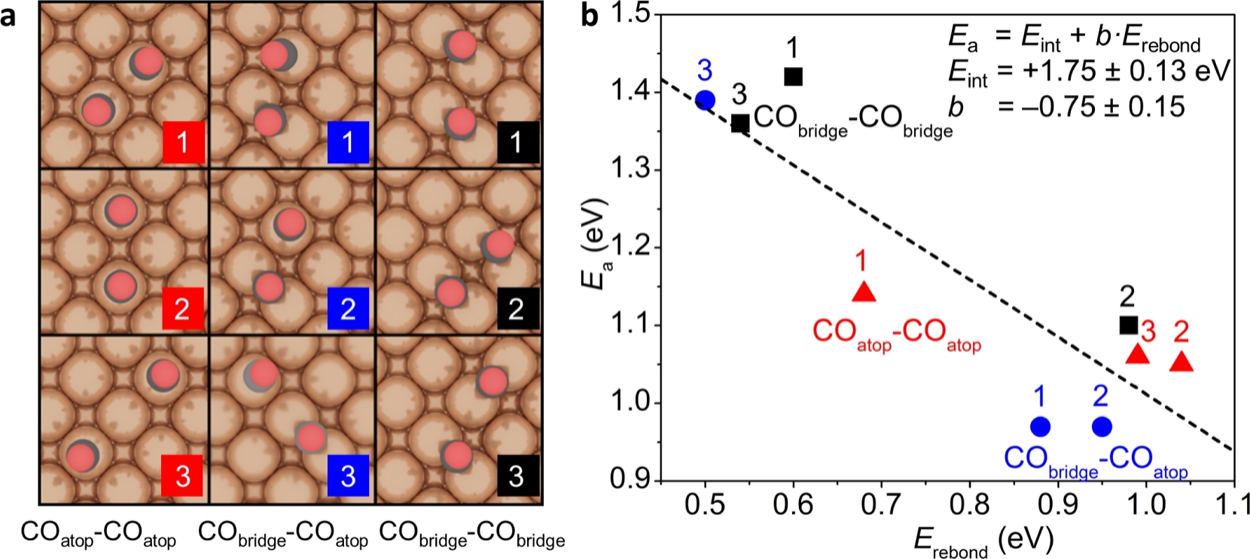
C–C coupling geometries depending on the CO adsorption configuration. (a) Adsorption geometry for different CO_atop_–CO_atop_ (left), CO_bridge_–CO_atop_ (center), and CO_bridge_–CO_bridge_ (right) precursors. (b) Activation barriers *E*_a_ for C–C coupling from different CO_atop_–CO_atop_ (red), CO_bridge_–CO_atop_ (blue), and CO_bridge_–CO_bridge_ (black) precursors *vs* rebonding energy, *E*_rebond_ = E_*CO(1)-ts_ + E_*CO(2)-ts_ (eqs 1 and 2).^45^E_*CO(1)-ts_ (E_*CO(2)_) is the adsorption energy of one CO molecule in the CO–CO transition state (ts) geometry once the other CO is excluded. The number labels relate each data point in panel (b) to the configuration of its initial state in panel (a).

To reveal the catalytic role of the CO binding configuration in the formation of C_2+_ products, we calculated C–C coupling on Cu(100) from CO precursor pairs bound in atop and bridge configuration (see Computational Details in the Supporting Information). Our theoretical and experimental spectroscopic results show a direct correlation between high surface coverage of weakly bound CO_atop_ at the catalyst surface (0.75 ML, determined *via* DFT, Figure 3c) and C_2+_ production (detected experimentally, Figure 1f). In the literature, CO_atop_ hydrogenation was reported more favorable than the reduction of bridge-bound CO,^16^ and a lower activation barrier for CO–CO dimerization was calculated for a CO_atop_–CO_bridge_ precursor rather than CO_bridge_–CO_bridge_.^4^ C–C activation barrier is a key descriptor for assessing C_2+_ selectivity since CO–CO coupling to form the OCCO^–^ dimer is considered the rate-determining step for CO_2_ electroreduction to C_2+_ products.^46^ Thus, we assessed the thermodynamics and kinetics of this process from nine different coupling configurations at θ_*CO_ = 0.11 ML (Figures 4a and S34) to rationalize the facile CO_atop_ reduction to C_2+_ at −1.05 V_RHE_ (Figure 1f). In general, our calculations show that the formation of the OCCO^–^ dimer from CO_atop_–CO_atop_(CO_bridge_) precursors (red and blue data points in Figure 4b) has an activation barrier around 0.9–1.0 eV, ∼0.4 eV lower than its formation from the lower-coverage CO_bridge_–CO_bridge_ precursor (1.4 eV, black data points in Figure 4b). This means that C_2+_ products are more easily formed if at least one CO_atop_ participates in the critical CO–CO dimerization step.

Since standard Bronsted–Evans–Polanyi-type linear scaling relationships between the Gibbs free energy^47^ and the activation energy of C–C coupling cannot account for this 0.4 eV difference between atop and bridge adsorption configurations, we employed the Hammer’s decomposition scheme for the activation energies.^45,48^ Hammer’s formalism states that the activation energy for dissociation (coupling) processes correlates with *E*_rebond_, the rebonding energy required to bind products (dissociation) or rebind precursors (coupling) from the transition-state configuration. This correlation involves as well a geometric offset, *E*_int_, which describes the interaction (repulsion) between precursors. Thus, the activation barriers for C–C coupling from the nine different CO_*x*_–CO_*y*_ initial configurations scale with the energy to rebind the CO precursors (Figure 4b, eq 1), here calculated as adsorption energies of each CO molecule in the absence of the coupling partner (eq 2).
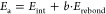
1
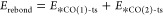
2

Adsorption configurations which account for less endothermic rebonding energies (*E*_rebond_ ∼ +0.5 eV, Figure 4b), such as bridge–bridge, can rebind CO molecules from the transition state, thus impeding a successful C–C coupling. Instead, atop–atop(bridge) configurations show poor interaction with the transition state (very endothermic *E*_rebond_ ∼ +1.0 eV, Figure 4b), which hinders CO–CO dissociation and enables the formation of the final state, *OCCO^–^. The promoting effect of atop–atop(bridge) adsorption configurations is further confirmed by the structural and electronic features of the transition-state geometry. C–O distance, *d*_C–O(2)_ = 1.26 Å, and OCCO Bader charge, *q*_CO–CO_ = −0.9 |e^–^| are close to the characteristics of *OCCO^–^ (*d*_C–O(2)_ = 1.39 Å, *q*_OCCO_ = −1.0 |e^–^|, Tables S7 and S8), thus suggesting a fast evolution toward the dimer. Finally, the interaction energy, *E*_int_, accounts for a positive offset, 1.75 ± 0.13 eV, due to the repulsion between the CO fragments (Figure 4b).

As a final remark, both, experimental results and theoretical insights confirmed that CO coverage is the key in enabling C–C coupling on Cu and providing a molecular-level understanding of the change of the F.E. of C_2+_ products *versus* CO at different potentials. High CO coverage at an appropriate potential (−1.05 V_RHE_) implies large surface population of C–C selective CO_atop_ (Table S2) and reduces the occurrence of the competing hydrogen evolution reaction (minimum F.E. at about −1.0 V_RHE_). Instead, lower CO coverages at more positive or negative potentials affect CO_2_ reduction toward ethylene negatively since CO_atop_ can convert to more static configurations (*e.g.*, CO_bridge_),^16,53^ either inert for CO_2_ reduction,^16^ or precursors for methane formation, in agreement with our evidence of high CH_4_ F.E. at −1.15 V_RHE_ (Figure S8).^49^

**Figure 5:**
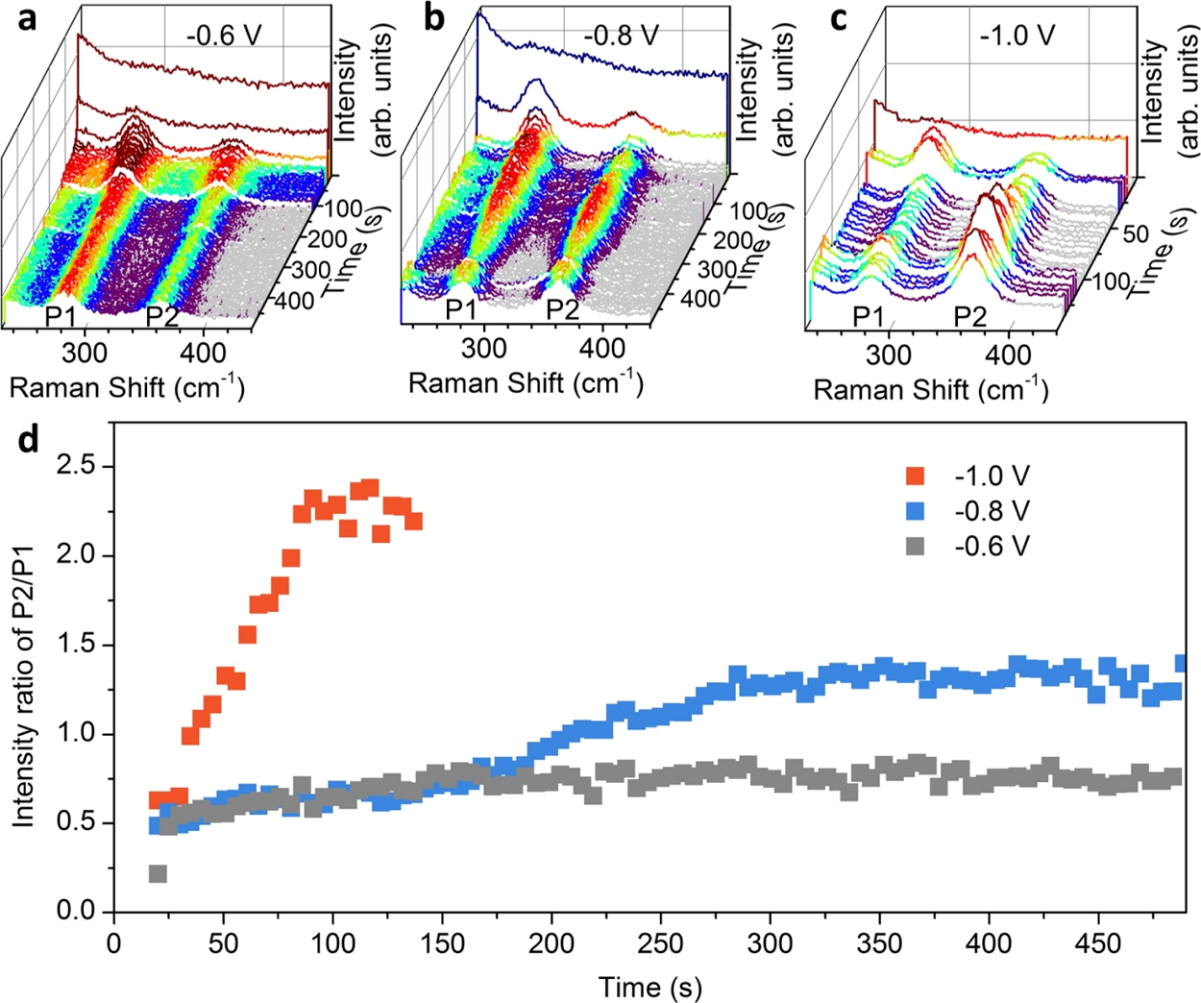
Time-dependent *operando* Raman spectra acquired on Cu_2_O nanocubes at different potentials during CO_2_RR. (a) −0.6 V_RHE_. (b) −0.8 V_RHE_. (c) −1.0 V_RHE_. (d) Time dependence of the peak ratio at different applied potentials vs RHE.

Furthermore, we applied *operando* Raman spectroscopy to track the temporal evolution of the surface CO coverage on Cu during the CO_2_RR. Figure 5 shows time-dependent Raman spectra with a resolution of 5 s acquired at different applied potentials, −0.6 V_RHE_, −0.8 V_RHE_, and −1.0 V_RHE_ (Figure 5a,b,c respectively), corresponding to the potential of CO formation, the initial potential of CO conversion, and the optimal potential of C–C coupling during CO_2_RR, respectively. No significant surface adsorption of CO was detected in the first 5 s due to the reduction of Cu_2_O, and Figure 5d shows the data after 20 s (potentials were applied after 15 s). At −0.6 V_RHE_, CO generated by CO_2_RR adsorbs on the Cu surface and reaches a steady state within 150 s. The time-dependent curve mainly reflects the balance of the CO generation with CO desorption and adsorption. The surface concentration of CO increases rapidly due to the large number of accessible Cu active sites at the beginning of the reaction and then reaches equilibrium as the number of available active surface sites decreases and the adsorption and desorption are balanced. At −0.8 V_RHE_, the CO coverage increases faster than at −0.6 V_RHE_ to reach a similar equilibrium of the peak ratio at about 0.8, which means faster CO formation rate. However, there is a second-wave increase of the CO surface coverage after ∼180 s. The most reasonable explanation is the existence of another adsorption site or configuration of CO on the electrode surface. With the saturation of the first type of adsorption site, CO gradually adsorbs on another site with lower adsorption free energy. DFT simulations confirmed these experimental results (Table S2): for θ_CO_ ≥ 0.6 ML, CO_atop_ population increases and CO binding energy decreases to 0.2–0.3 eV, fingerprint of weakly bound CO_atop_, with the C=O rotation band (P1) less intense (Table S4). Thus, the time-dependent curve of the peak ratio shows a process of equilibrium in two phases. At a more negative potential (−1.0 V_RHE_), the optimal potential for C–C coupling during CO_2_RR, the CO coverage increases more quickly and reaches a higher equilibrium coverage, which determines a higher P2/P1 ratio. Although the complex CO_2_RR mechanism and the electrode structure made it difficult to give a kinetics equation, we provide an effective strategy to investigate the dynamics of CO on the Cu surface during the CO_2_RR.

## Conclusions

In conclusion, *operando* Raman spectroscopy and DFT modeling were used to reveal the change of the electrode structure and the composition and dynamics of the surface intermediates during CO_2_RR on Cu_2_O nanocubes. During the CO_2_RR, a Raman band at about 360 cm^–1^ appeared between +0.3 and 0.0 V_RHE_, which was assigned to surface copper carbonate species formed from the KHCO_3_ electrolyte. We also revealed that the ratio of the Cu–CO stretching (P2) and Cu–CO rotation (P1) bands changes with the applied potential and is strongly related to the CO coverage, allowing us to track the dynamics of the CO surface coverage on Cu during CO_2_RR. Interestingly, a clear correlation exists between the P2/P1 ratio and the F.E. of the C_2+_ products on the applied potential. Our experiments and theoretical insights allowed us to conclude that the degree and ease of the C–C coupling is determined by the CO surface coverage, which in turn influences the preferred CO adsorption configuration. At a high surface coverage, CO adsorbs in a mix of atop and bridge sites, and both CO_atop_–CO_atop_ and CO_atop_–CO_bridge_ couplings are thermodynamically and kinetically more favorable than CO_bridge_–CO_bridge_ due to both electronic and structural effects. Weakly bound atop configurations show no interaction with CO in the transition state, thus limiting CO–CO dissociation and boosting the evolution toward the final state OCCO^–^. Overall, we were able to provide molecular-level insight into the correlation between the CO coverage and the potential-dependent C_2+_ F.E. Finally, it was illustrated that *operando* Raman is an effective method to investigate the electrochemical solid–liquid interface and the interaction of surface intermediates with the electrode during an electrocatalytic reaction.

## Supporting Information Available

Datasets generated during the current study are available in the ioChem-BD database^50^ at DOI: 10.19061/iochem-bd-1-192. Synthesis of Cu_2_O cubes and electrodes, *ex situ* and quasi-*in situ* characterization of samples, *operando* Raman experiments, CO_2_RR testing, and computational details (PDF)
