# Supplementary material for: Revealing the CO Coverage-Driven C–C Coupling Mechanism for Electrochemical CO2 Reduction on Cu2O Nanocubes via Operando Raman Spectroscopy
Source: ACS Catal. 2021 Jun 11;11(13):7694–701. doi: 10.1021/acscatal.1c01478 (PMC8256421; doi:10.1021/acscatal.1c01478)
Supplement: Supplementary file 1 — cs1c01478_si_001.pdf [file cs1c01478_si_001.pdf]

Supporting Information

Revealing the CO Coverage Driven C-C Coupling Mechanism for Electrochemical CO_2 Reduction on Cu_2O Nanocubes *via Operando* Raman Spectroscopy

Chao Zhan[1], Federico Dattila[2], Clara Rettenmaier[1], Arno Bergmann[1], Stefanie Kühl[1], Rodrigo García-Muelas[2], Núria López[2*], Beatriz Roldan Cuenya[1*]

[1]Department of Interface Science, Fritz-Haber Institute of the Max-Planck Society, Faradayweg 4-6, 14195 Berlin, Germany.
[2]Institute of Chemical Research of Catalonia (ICIQ), The Barcelona Institute of Science and Technology (BIST), Av. Països Catalans 16, 43007 Tarragona, Spain.

e-mail: nlopez@iciq.es; roldan@fhi-berlin.mpg.de

Methods:
Synthesis of Cu_2O cubes and Electrodes

Cu_2O nanocubes (NCs) were synthesized via a surfactant free protocol by using Ascorbic Acid as reductant.[1] In a typical synthesis, 4 ml 0.1 M $CuSO_4$ (Sigma-Aldrich, >98%) were diluted in 366 ml H_2O and stirred vigorously. Then 14 ml 1 M NaOH (Alfa Aesar, >97%) were added to start the nucleation process. After 10 s, 16 ml of 0.25 M L-Ascorbic Acid (Sigma Aldrich, reagent grade) was added to start the reduction and NC growth. The solution was stirred further for 13 min. The obtained sample was washed three times with a EtOH:H_2O mixture (1:1) and two times with EtOH. The clean sample was stored in 20 ml of EtOH. ICP-MS was performed on the sample after digestion in HNO_3 and HCl and revealed a Cu concentration of 1.2 mg/ml.

The electrodes were prepared by drop-casting 50 µl on glassy carbon (2.5 cm × 1 cm) or drop-casting 40 µl on both sides of 1 cm^2 carbon paper (Toray Carbon Paper, GGP-H-60). As electrolyte, 0.1 M $KHCO_3$ (Alfa Aesar, 99.7-100.5%) was used after purification with a cation-exchange resin (Chelex 100 Resin, Bio-Rad).

Ex-situ and Quasi *in situ* Characterization of samples

The crystal structure of the catalysts was studied using X-ray diffraction using a Bruker-AXS D8 Advance in Bragg-Brentano configuration equipped with an energy-dispersive position-sensitive LynxEye detector and Cu X-ray tube. The sample was deposited on a low-background Si substrate and the diffraction pattern recorded between 20 and 90° with a step-size of 0.02° and 13 s. The X-ray diffraction pattern were analysed using Rietveld refinement with the software package Topas (v6, Bruker-AXS). The Cu_2O phase (space group: Pn3m) was fitted using the scale factor, the lattice parameter *a*, the size-induced peak broadening, a preferred orientation of the Cu_2O nanocubes on the flat substrate using the spherical harmonics corrections as well as the zero error of the 2θ scale, sample displacement, and the instrumental peak broadening of the diffractometer.

The size and morphology of the samples were determined by Transmission Electron Microscopy (TEM) with a FEI Talos F200X microscope and by Scanning Electron Microscopy (SEM) with a Thermo Fisher Apreo microscope. TEM samples were prepared by placing a drop of the as-synthesized nanocube solution or a solution containing the reacted nanocubes after CO_2RR (obtained after removal from the electrode by a sonication treatment in 150µl ethanol) on a nickel grid (400 mesh with lacey carbon film, PLANO GmbH) and allowing it to dry in air.

XPS measurements were performed with a commercial Phoibos100 analyser (SPECS GmbH, E_{pass} = 15 eV) and a XR50 (SPECS GmbH) X-ray source with an Al anode ($E_{K\alpha}$=1486.7 eV). All spectra were aligned by fixing the Cu $2p_{3/2}$ of Cu^0 and Cu^+ to 932.67 eV as reference and fitted using a Shirley-type or a linear background subtraction for X-ray or Auger electron spectroscopy, respectively. Quasi *in situ* XPS experiments were performed while avoiding the exposure of the sample to air after the electrochemical treatment. In this setup, an electrochemical cell is directly attached to

the ultrahigh vacuum (UHV) system where the XPS chamber is located to allow the sample transfer without air exposure (Figure S7). The electrochemical measurements were carried out using a potentiostat (Autolab PGSTAT 302N).

Operando Raman experiments

The *operando* Raman spectra were obtained by means of a Renishaw (InVia Reflex) confocal Raman microscope with a 785 nm laser. To perform the *operando* experiments in an electrolyte, a water immersion objective with a long working distance (Leica microsystems, 63x, numerical aperture of 0.9) was chosen. The objective with a long working distance is needed to avoid diffusion hindrance problems during the Raman measurements. The laser power was about 0.36 mW. The acquisition time was 10 s for the steady-state experiments at different potentials and 5 s for the time-dependent experiments. For the *operando* measurements, the objective is protected from the electrolyte by a Teflon film (DuPont, film thickness of 0.013 mm). A drop of water is used to drive away the air between the film and the objective to match the refractive index, which ensures efficient excitation and collection of the Raman signal. The electrochemical measurements were performed in a home-built spectro-electrochemical cell made of Teflon and controlled by a Biologic SP-240 potentiostat (Figure S10). The cell was equipped with a reference electrode (leak-free Ag/AgCl, Alvatek), a counter electrode (Pt ring), and a working electrode with the catalyst drop-casted on glassy carbon. Typically, a 15 ml CO_2-saturated 0.1 M $KHCO_3$ solution was used as electrolyte, and CO_2 was continuously injected into the solution during the experiment. Ar-saturated 0.1 M $KHCO_3$, Ar-saturated 0.1 M $NaClO_4$, CO-rich 0.1 M $KHCO_3$ were used in the experiments as well. We mixed the CO-saturated 0.1 M $KHCO_3$ solution with the Ar-saturated 0.1 M $KHCO_3$ solution to prepare the 33% CO-rich 0.1 M $KHCO_3$ and 66% CO-rich 0.1 M $KHCO_3$. The percentage represents the volume fraction of CO-saturated $KHCO_3$ in the electrolyte. For the steady-state experiment, each potential was applied for at least 10 min before collecting the spectra to ensure steady-state conditions at the surface of the catalyst. For the time-dependent experiment, we acquired three spectra at open circuit potential, then applied the potential and continuously recorded the Raman spectra every 5 s.

CO_2RR testing

Electrocatalytic measurements were performed with an Autolab (Metrohm) potentiostat in a H-type cell equipped with an anion exchange membrane (Selemion AMV, AGC Inc). The three-electrode system consisted of the catalyst deposited on carbon paper as working electrode, a platinum gauze (MaTecK, 3600 mesh cm^{-2}) as counter electrode, and a leak-free Ag/AgCl reference electrode (LF-1, Alvatek). A 0.1 M $KHCO_3$ aqueous solution was used as electrolyte and saturated with CO_2 (99.995%) for at least 20 min prior to the measurements. The CO_2 flow was 20 ml/min. The electrochemical protocol consisted of a linear sweep voltammogram from the open circuit potential to the cathodic potential followed by chrono-amperometry at this

potential for 3600 s. All potentials are given versus the RHE scale and were corrected for the iR drop. Each presented data point corresponds to an identical freshly prepared sample following this protocol at different potentials. The electrochemical surface roughness factor was estimated from double layer capacitance measurements.[2]

Gas products were detected and quantified every 15 min by online Gas Chromatography (GC, Agilent 7890B), equipped with a Thermal Conductivity Detector (TCD) and a Flame Ionization Detector (FID). Liquid products were analyzed after each measurement with a high-performance liquid chromatograph (HPLC, Shimadzu Prominence), equipped with a NUCLEOGEL SUGAR 810 column and a refractive index detector (RID), and a liquid GC (L-GC, Shimadzu 2010 plus), equipped with a fused silica capillary column and a FID detector.

All catalytic results in this study are shown in terms of Faradaic efficiency (F.E.). The Faradaic efficiency of the gas product *x* was calculated as:

$$F.E. = \frac{\dot{V} \times C_x \times z_x \times F}{A \times V_\mathrm{M} \times j_\mathrm{total}} \times 100\%, \qquad \text{Eq. (1)}$$

and for the liquid product *x* was calculated as

$$F.E. = \frac{V \times \Delta C_x \times z_x \times F}{\Delta Q} \times 100\%. \qquad \text{Eq. (2)}$$

Here

$F.E$: Faradaic Efficiency of product x.

$\dot{V}$: CO_2 gas flow rate / l s^{-1}.

C_x: Volume-fraction of the product x detected by GC.

z_x: Electrons transferred for reduction to product x.

F: Faradaic constant / C mol^{-1}.

A: Geometric area of the electrode / cm^{-2}.

V_M: Molar volume / 22.4 l mol^{-1}.

j_total: Total current density during CO_2 bulk electrolysis / A cm^{-2}.

ΔC_x: Final concentration of product x detected by HPLC and liquid GC / mol l^{-1}.

ΔQ: Total charge transferred during electrolysis at const. potential or current/C.

V: Volume of the electrolyte / l.

Computational Details

The Density Functional Theory (DFT) calculations were conducted using the PBE density functional[3] within VASP[4,5]. To account for interactions between catalyst surface and adsorbates, we included dispersion through the DFT-D2 method,[6,7] with our re-parametrization of the C_6 coefficients for metals.[8] CO adsorption energies were corrected for implicit solvation contributions within the VASP-MGCM framework.[9,10] Inner electrons were represented by PAW pseudopotentials[11,12] and we expanded the monoelectronic states for the valence electrons as plane waves with a kinetic energy cutoff of 450 eV. We sampled the Brillouin zone by a Γ-centered k-point mesh from the Monkhorst-Pack method,[13] with a reciprocal grid size smaller than 0.03 Å^{-1}. Hubbard corrections were applied *via* the Dudarev approach[14] to the 2p orbitals of C and O to

tune the HOMO-LUMO gap of the CO molecule. The U parameter was varied between 1.25 and 2.00 eV, whilst J was always kept equal to 1 eV. The resulting $U_{eff} = U - J$ parameter was then in line with DFT studies on site preferences on Cu(100) (Figure S31).[15] To assess electric field effects (Figure S32), we employed the corresponding tag in VASP which introduces it *via* a dipole correction.[8,16]

We modeled the oxide-derived Cu catalysts as Cu(100) slabs at least four layers thick. The two outermost layers were fully relaxed to allow surface reconstruction, whilst the rest fixed to mimic the bulk. The vacuum between the slabs was always larger than 10 Å. Different surface structures were employed to study CO surface coverage, θ_{CO}, between 0.11 and 0.88 ML,[17] Table S2, and the initial CO configurations were retrieved from a theoretical study on high CO coverage adsorption configurations on Pt(100).[18] CO molecules were placed only on one side of the slab, thus, we applied a dipole correction to remove spurious artifacts from the asymmetric slab model.[19] To benchmark spectroscopic experimental evidences, we calculated the vibrational modes for the lowest energy CO adsorption configuration at different surface coverages, Table S2. We then determined the intensity ratio of the Cu-CO stretching band and CO restricted rotation from the superposition of these vibrational modes associated to different CO adsorption sites (Figure S33, Table S4-S6). To calculate CO-CO activation barrier, we employed a simplified computational setup with low CO coverage, implicit solvation,[9] and no electric field applied. In general, coverage and electric field effects are reported to stabilize CO-CO dimerization on Cu(100) by 0.2 eV and 1.0 eV, respectively,[20-22] and both factors affect CO_{bridge} and CO_{atop} configurations equally (Figure S32). Thus, our simplified approach does not alter the overall trend among different adsorption sites. Transition states for C-C coupling were located through the Climbing Image Nudged Elastic Band (CI-NEB) method and all of them exhibit a single imaginary vibrational frequency.[23] CO adsorption energies and kinetic barriers at θ_{CO} = 0.11 ML were reported using as references: CO_2(g), H_2(g), and the pristine Cu surfaces, in line with the Computational Hydrogen Electrode formalism.[24,25] Gibbs free energies (G) were calculated at 298.15 K by correcting DFT energies (E) for entropic effects.

Figures:

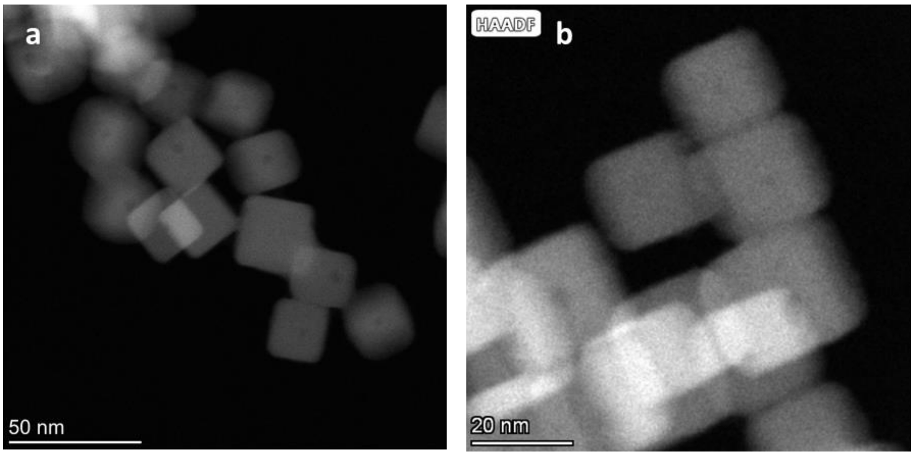

Figure S1. HAADF-STEM images of as-prepared Cu_2O nanocubes.

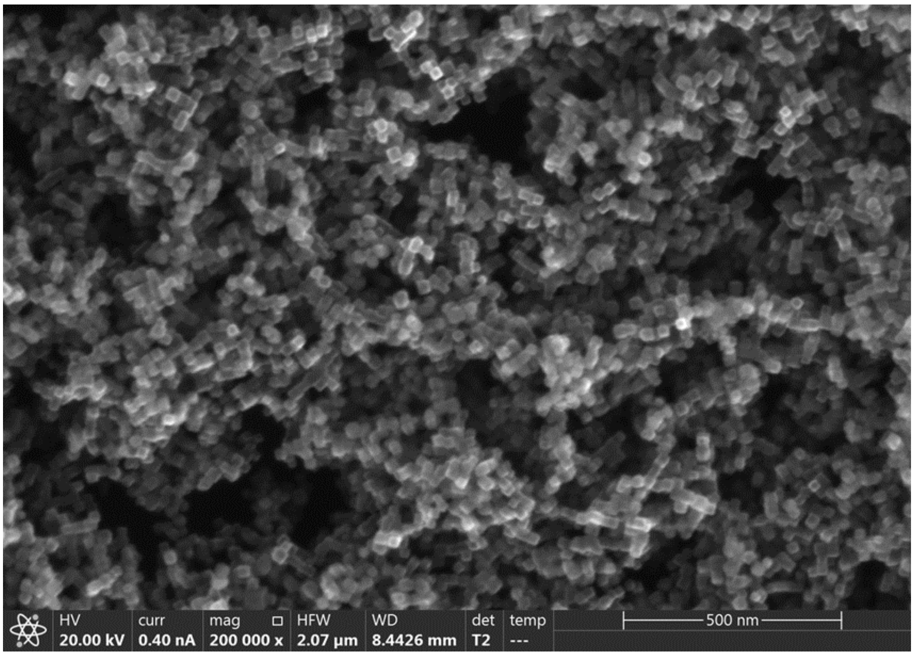

Figure S2. SEM image of the as-prepared Cu_2O nanocubes.

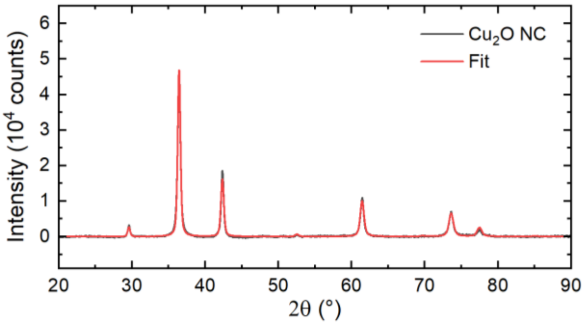

Figure S3. XRD pattern of the as-prepared Cu_2O nanocubes. We extracted the fit from Rietveld refinement using a Cu_2O phase (space group: Pn3m) leading to lattice parameter a = 4.267(2) Å and coherence length of 28.97(18) nm (determined from the integral breadth of the Bragg peaks). The fit exhibits a weighted-profile R of 2.07%.

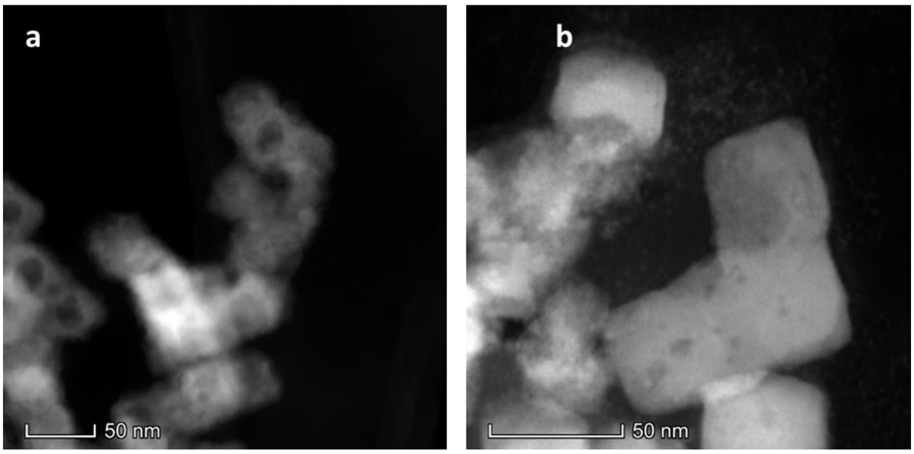

Figure S4. TEM images of the Cu_2O nanocubes after the CO_2RR in CO_2-saturated 0.1 M $KHCO_3$ electrolyte at –1.0 V_{RHE} for 1 h.

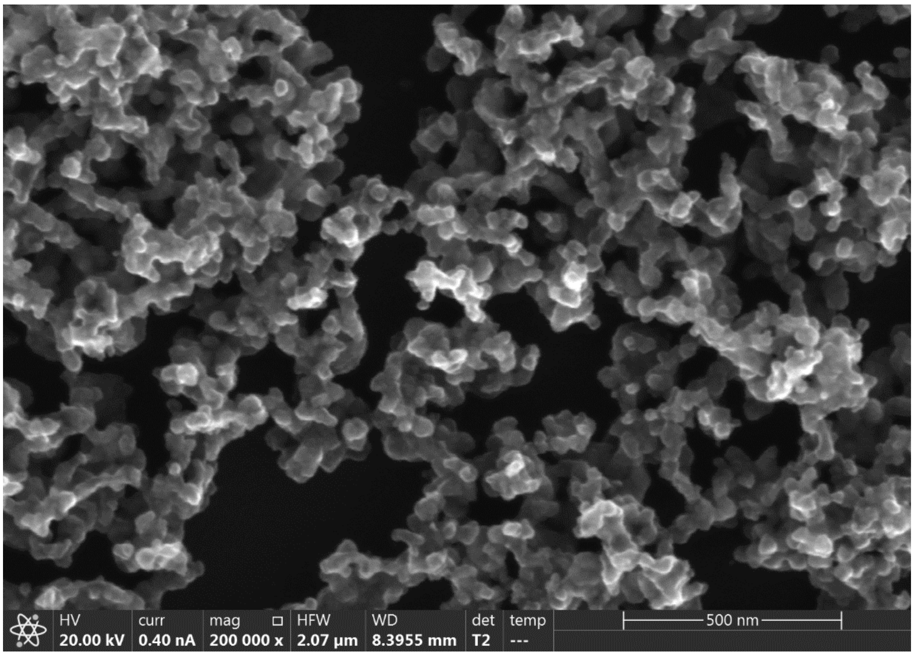

Figure S5. SEM image of the Cu_2O nanocubes after the CO_2RR in CO_2-saturated 0.1 M $KHCO_3$ electrolyte at –1.0 V_{RHE} for 1 h.

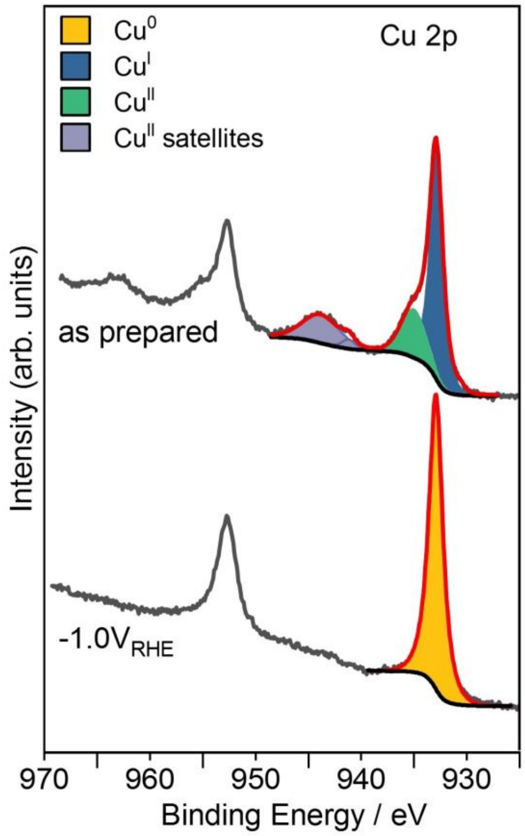

Figure S6. Quasi-*in situ* XPS spectra of the Cu 2p binding energy region of the as-prepared Cu_2O nanocubes and Cu_2O nanocubes after the CO_2RR at –1.0 V_{RHE} for 1 h in CO_2-saturated 0.1 M $KHCO_3$ electrolyte without air exposure with the corresponding fits for the Cu $2p_{3/2}$ orbital (red line). The Cu 2p core level region of the as prepared sample shows the presence of Cu^I (blue) and Cu^{II} (green) with its corresponding shake-up satellite (purple), while after 1h CO_2RR the species is attributed to Cu^0 (yellow).

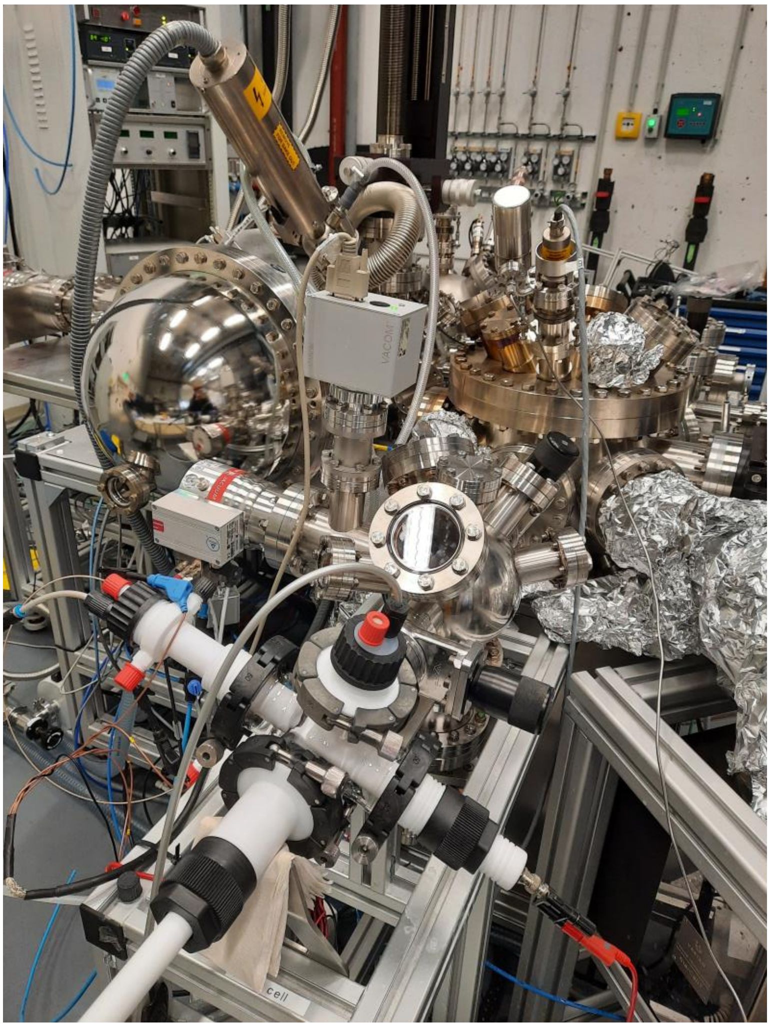

Figure S7. Photograph of the quasi-*in situ* XPS set-up, with the electrochemical cell directly interfaced to the UHV system.

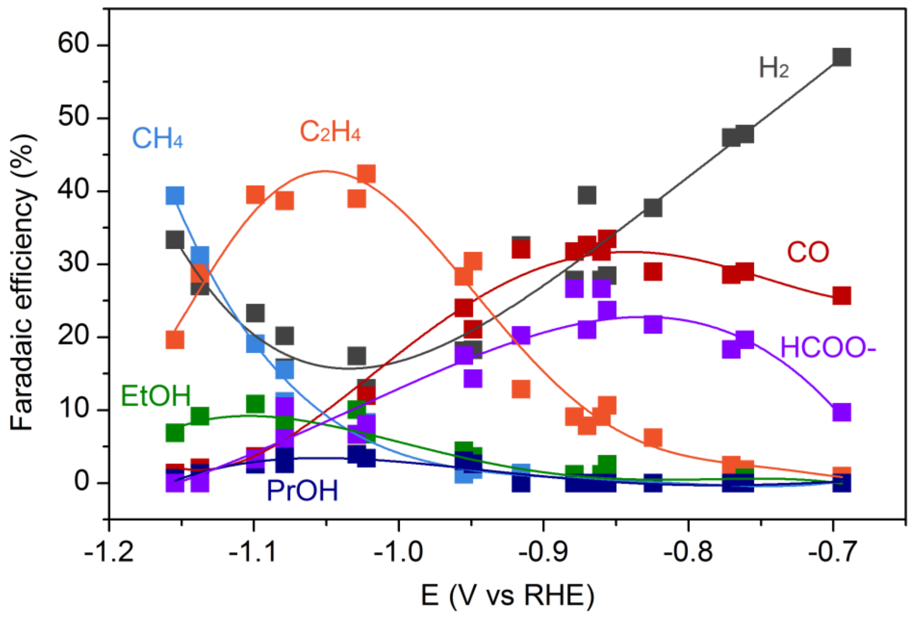

Figure S8. Faradaic efficiency for CO_2 reduction and hydrogen evolution reaction over Cu_2O nanocubes on carbon paper as a function of the applied potential obtained after 1h of CO_2RR in CO_2-saturated 0.1 M $KHCO_3$ electrolyte. Lines are included as guides for the eye.

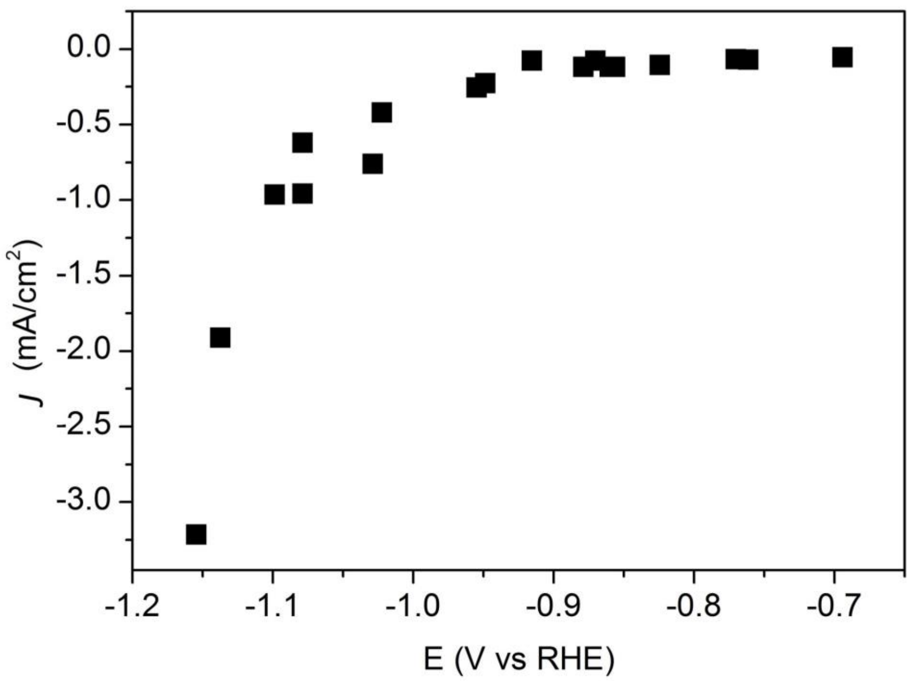

Figure S9. Current densities of Cu_2O nanocubes on carbon paper normalized to the electrochemical surface area as a function of the applied potential obtained after 1 h of CO_2RR. It indicates that the system is potential-controlled in the potential range presented. The catalyst surface roughness factor, estimated from double layer capacitance measurements, is 4.6.

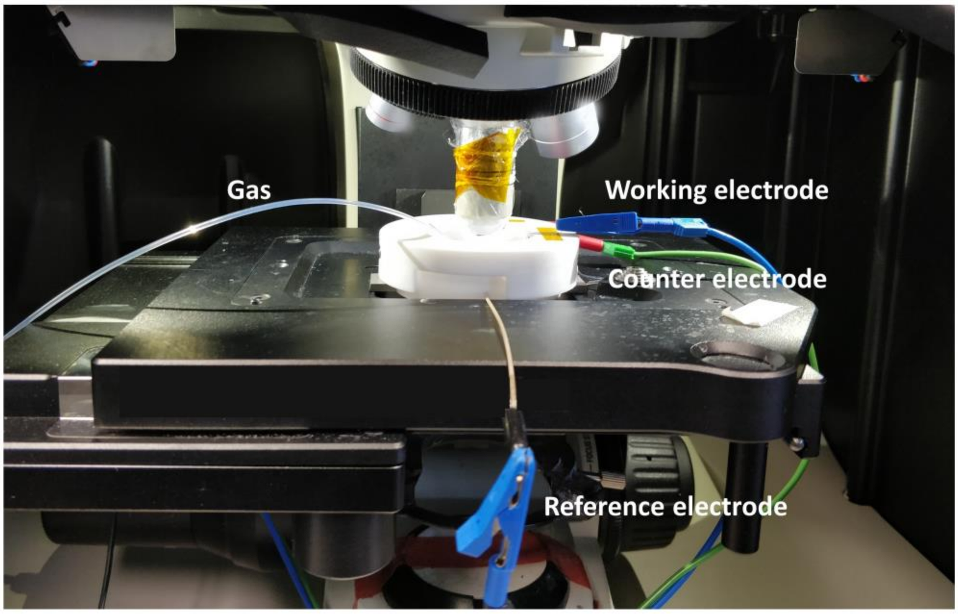

Figure S10. The photograph of the *operando* Raman set-up.

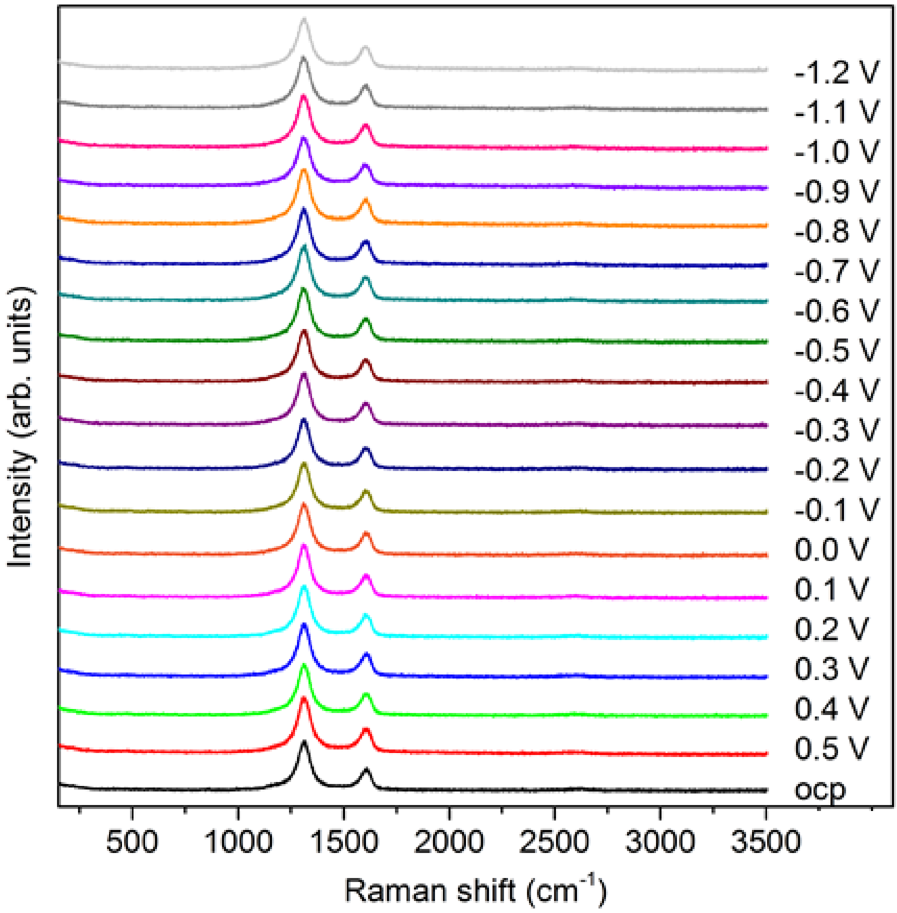

Figure S11. Raman spectra of the glassy carbon support in a CO_2-saturated 0.1 M $KHCO_3$ solution at different potentials. The glassy carbon support exhibits Raman peaks at 1313 cm^{-1} and 1616 cm^{-1}, corresponding to D- and G-Bands of graphitic carbon.[26]

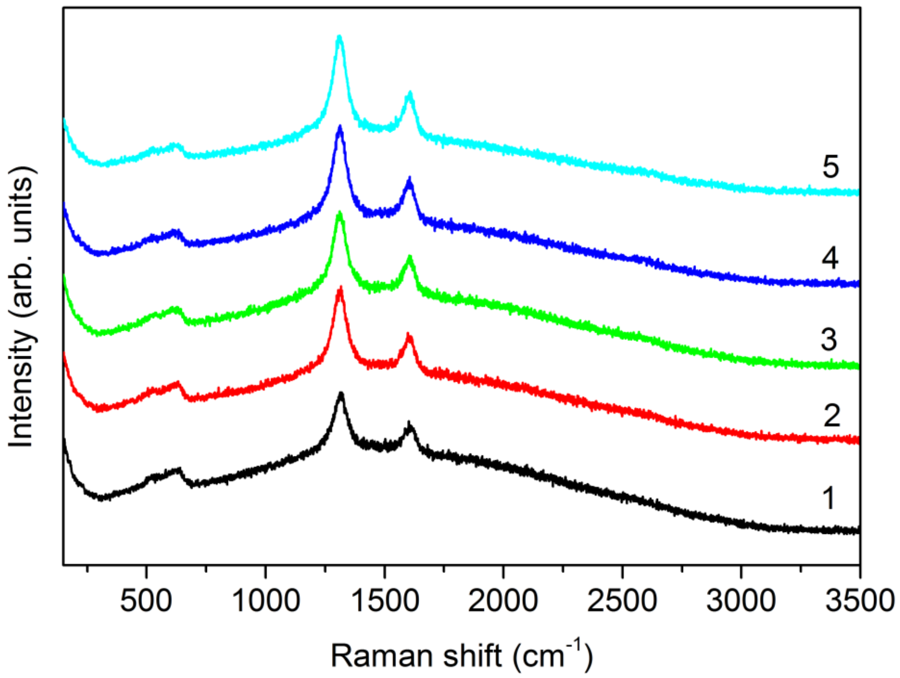

Figure S12. Consecutive Raman spectra acquired in a CO_2-saturated 0.1 M $KHCO_3$ solution at open circuit potential (OCP).

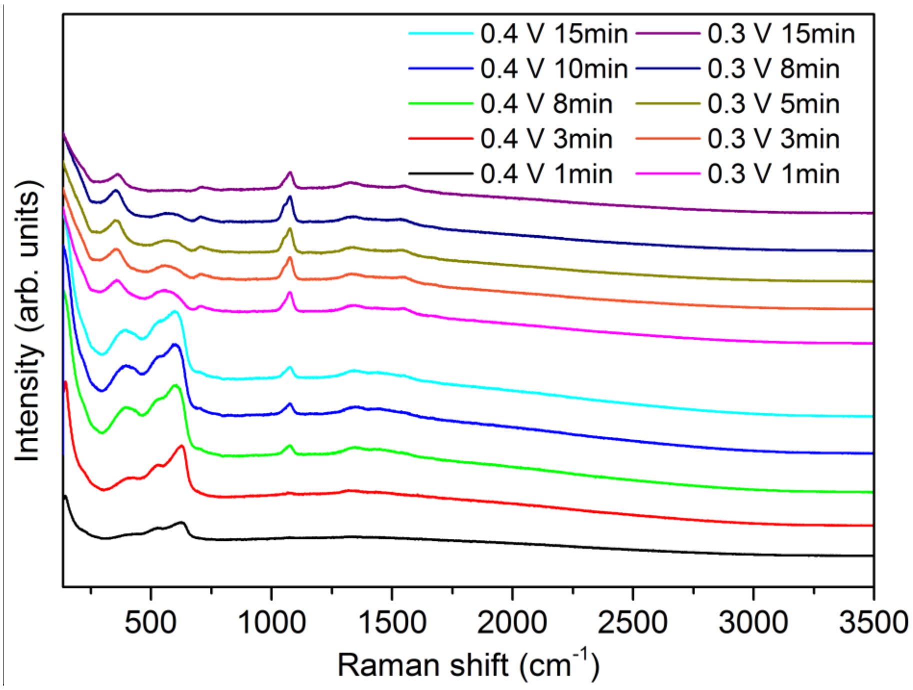

Figure S13. Time-dependent electrochemical surface-enhanced Raman spectra acquired during CO_2RR over Cu_2O cube catalysts at +0.4 V_{RHE} and +0.3 V_{RHE} in CO_2-saturated 0.1 M $KHCO_3$ solution. It can be found that the peak at about 360 cm^{-1}, which was at ~390 cm^{-1} at 0.4 V_{RHE}, co-exists with the peaks at 1077 cm^{-1} and 706 cm^{-1}, which are assigned to the carbonate species and surface hydroxyl species, respectively (Table S1).

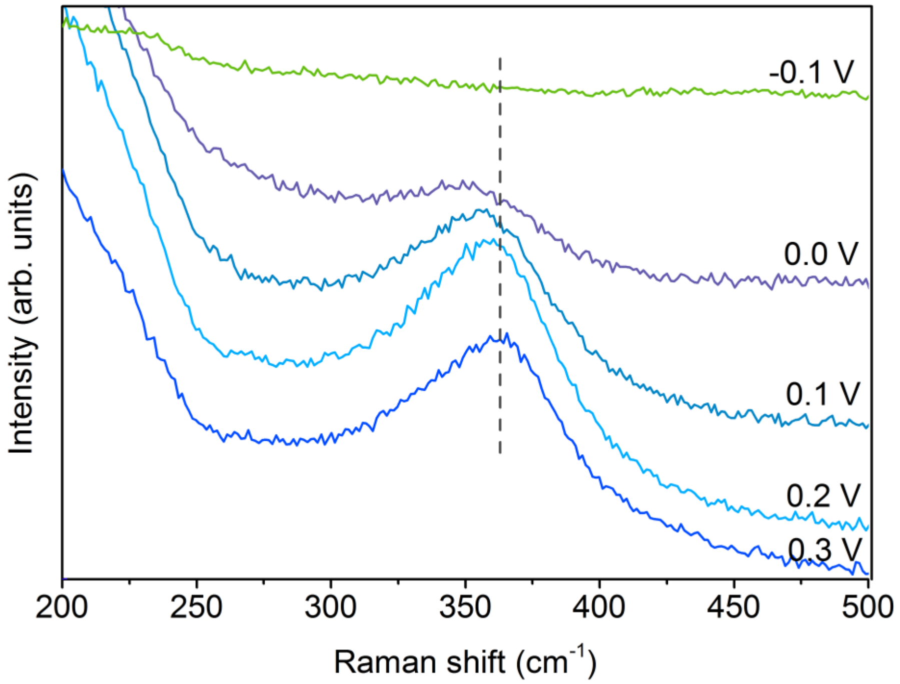

Figure S14. Zoom-in spectra of the Raman band at ~360 cm^{-1} acquired during CO_2RR in CO_2-saturated 0.1 M $KHCO_3$ from +0.3 to –0.1 V_{RHE}.

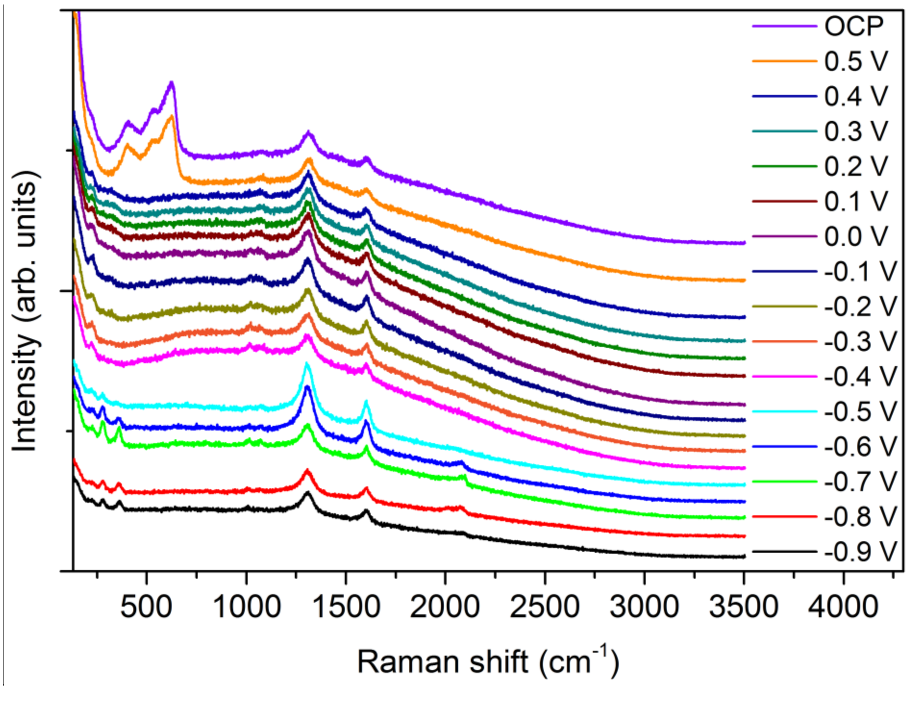

Figure S15. Electrochemical surface-enhanced Raman spectra of Cu_2O nanocubes during CO_2RR recorded at potentials from –0.9 V_{RHE} to OCP in a CO_2-saturated 0.1 M $KHCO_3$ solution.

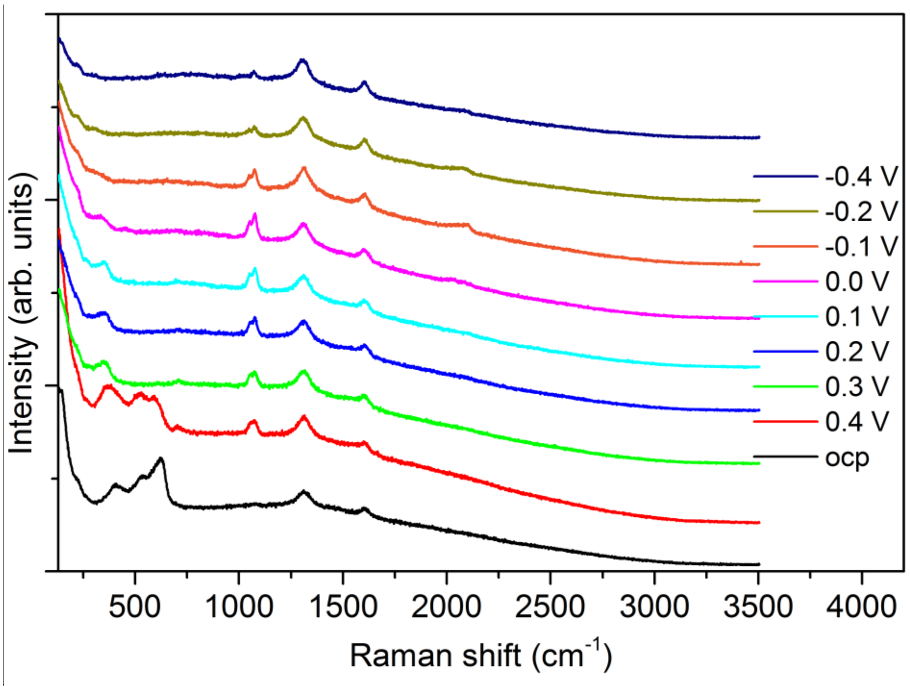

Figure S16. Electrochemical surface-enhanced Raman spectra of Cu_2O nanocubes during CO_2RR recorded at potentials from OCP to –0.4 V_{RHE} after scanning from a negative potential to a positive potential in a CO_2-saturated 0.1 M $KHCO_3$ solution.

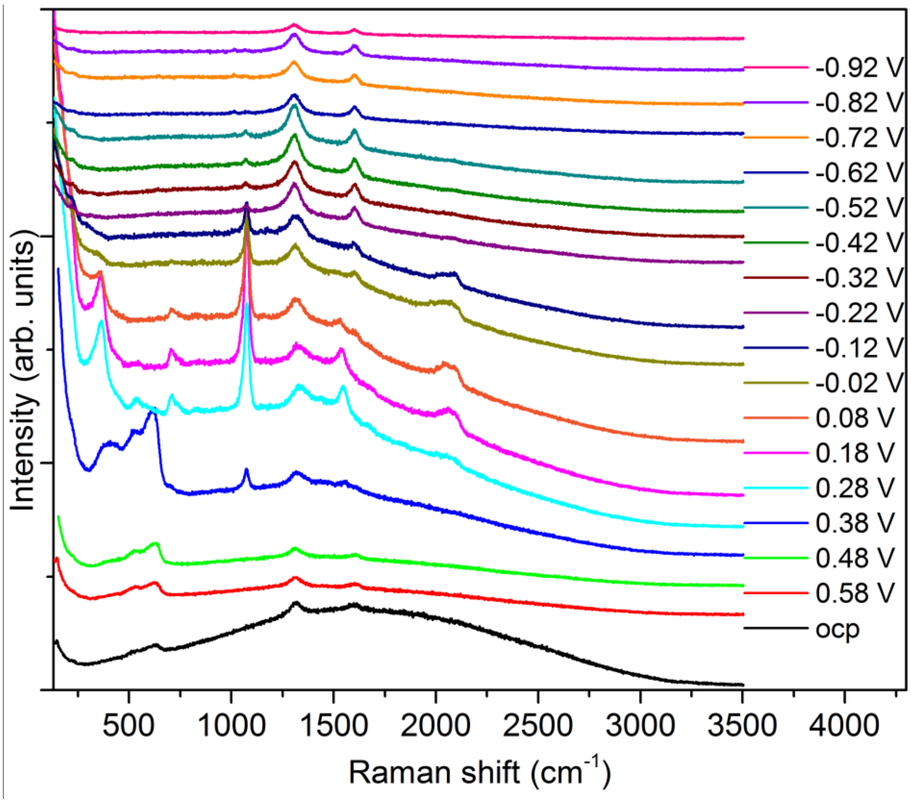

Figure S17. Electrochemical surface-enhanced Raman spectra of Cu_2O nanocubes recorded at potentials from OCP to –0.92 V_{RHE} in an Ar-saturated 0.1 M $KHCO_3$ solution.

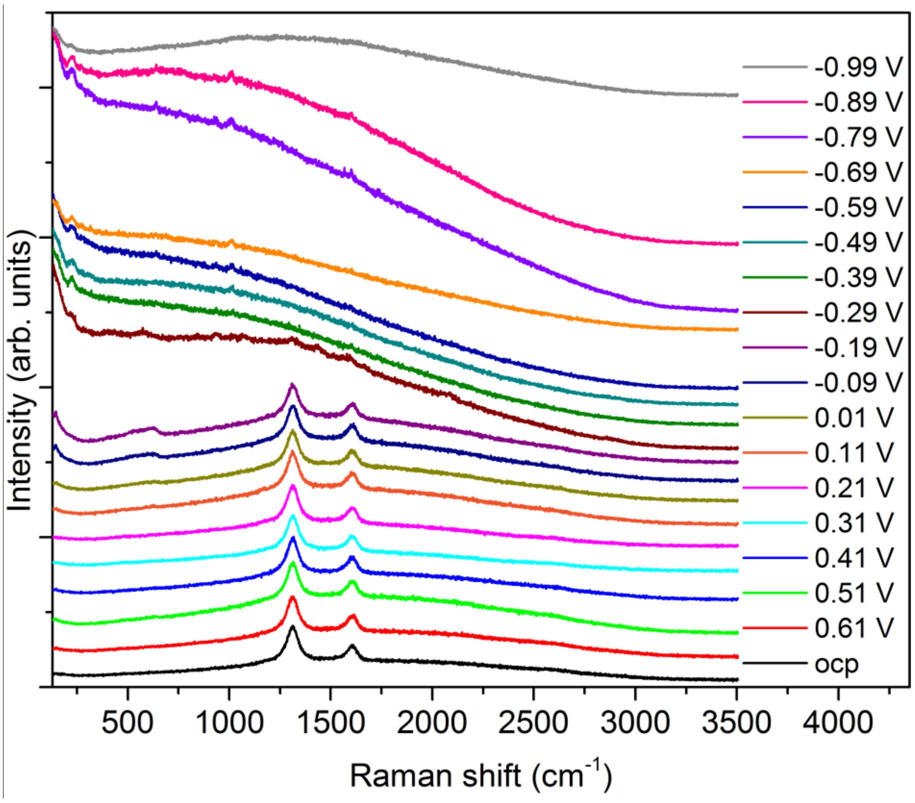

Figure S18. Electrochemical surface-enhanced Raman spectra of Cu_2O nanocubes recorded at potentials from OCP to –0.99 V_{RHE} in Ar-saturated 0.1 M $NaClO_4$ solution.

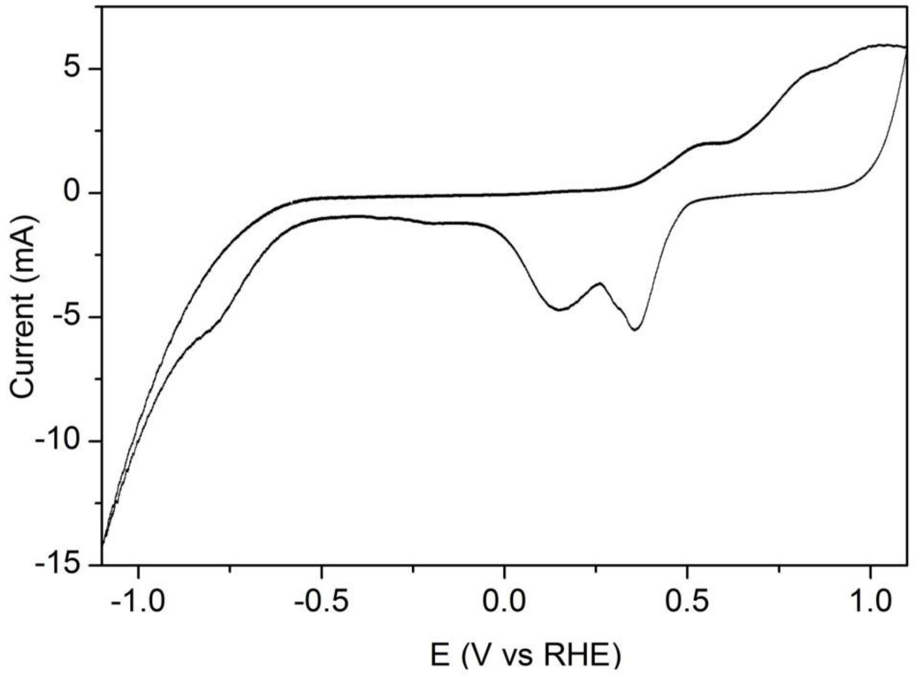

Figure S19. Typical cyclic voltammogram of Cu_2O nanocubes after their initial reduction in a CO_2-saturated 0.1 M $KHCO_3$ solution.

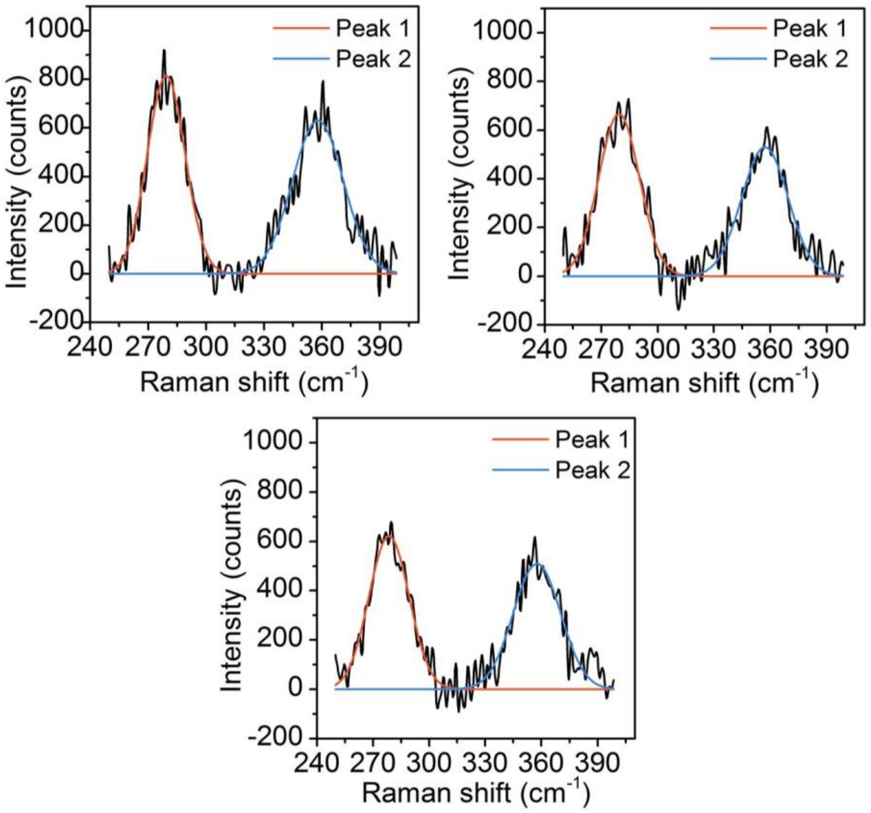

Figure S20. Typical fits of the CO rotation band (Peak 1) and Cu-C stretching band (Peak 2) at –0.6 V_{RHE} in a CO_2-saturated 0.1 M $KHCO_3$ solution.

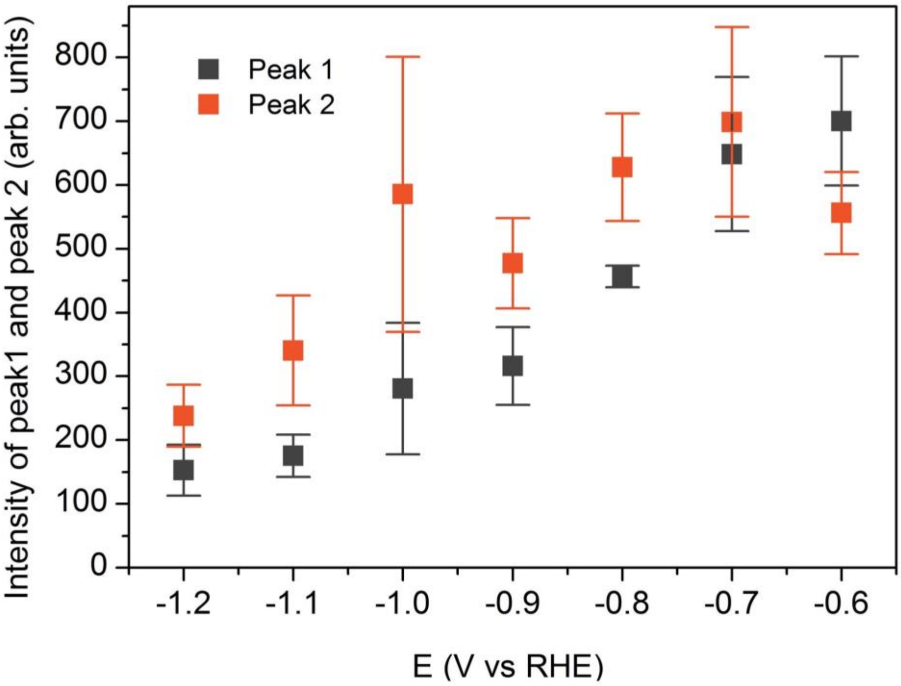

Figure S21. Raman intensity of Peak 2 and Peak 1 vs. the applied potential in a CO_2-saturated 0.1 M $KHCO_3$ solution. The trends of Raman intensity of Peak 2 and Peak 1 cannot match with the volcano trend of the CO_2RR Faradaic efficiency of multi-carbon products.

Individual error bars of P1 and P2 were obtained as the standard deviation and correspond to the average of three repeated measurements conducted on three identically prepared samples. The P2/P1 ratio was obtained as an average of the $(P2/P1)_i$ ratios extracted for i=1,2,3 different measurements. The uncertainty of the P2/P1 ratio was also defined as the standard deviation. The error bars of the average peak intensities (P1 or P2), Figure S21, coming from the intensity differences observed for each of these peaks in three different Raman spectra, are much larger than those of their average ratio (P2/P1), which was calculated by normalizing every $(P2)_i$ by the $(P1)_i$ from the same Raman spectrum (i). The error bar of the averaged P2/P1 was also obtained as the standard deviation and is shown in Figure 2c. Using the P2/P1 ratio instead of specific P2 and P1 intensities for comparison minimized the influence of the nanostructure-sensitive surface enhancement effect, which generally hampers the quantification of adsorbate surface concentrations (e.g. CO) directly from the spectral Raman intensity.

The formulas used to calculate error margins (standard deviation, SD) for the individual P1 and P2 and for the ratio R = P2/P1 are shown below, with i=1,2,3 indicating the three individual measurements conducted:

$$SD_{P1} = \sqrt{\frac{1}{2}\sum_{i=1}^{3}(P1_i - \mu_{P1})^2}\ , \qquad \mu_{p1} = \frac{1}{3}(P1_1 + P1_2 + P1_3)$$

$$SD_{P2} = \sqrt{\frac{1}{2}\sum_{i=1}^{3}(P2_i - \mu_{P2})^2}, \qquad \mu_{p2} = \frac{1}{3}(P2_1 + P2_2 + P2_3)$$

$$SD_R = \sqrt{\frac{1}{2}\sum_{i=1}^{3}(R_i - \mu_R)^2}\ , \qquad R_i = \frac{P2_i}{P1_i}, \qquad \mu_R = \frac{1}{3}(R_1 + R_2 + R_3)$$

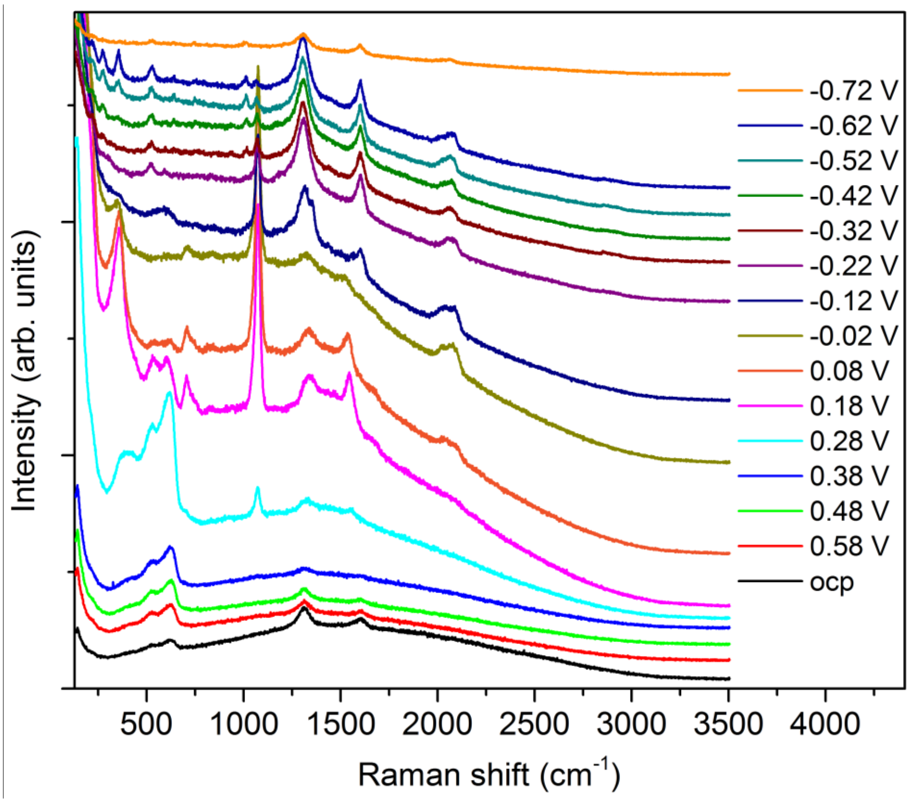

Figure S22. Electrochemical surface-enhanced Raman spectra of Cu_2O nanocubes acquired during CORR recorded at potentials from OCP to –0.72 V_{RHE} in CO-saturated 0.1 M $KHCO_3$ solution.

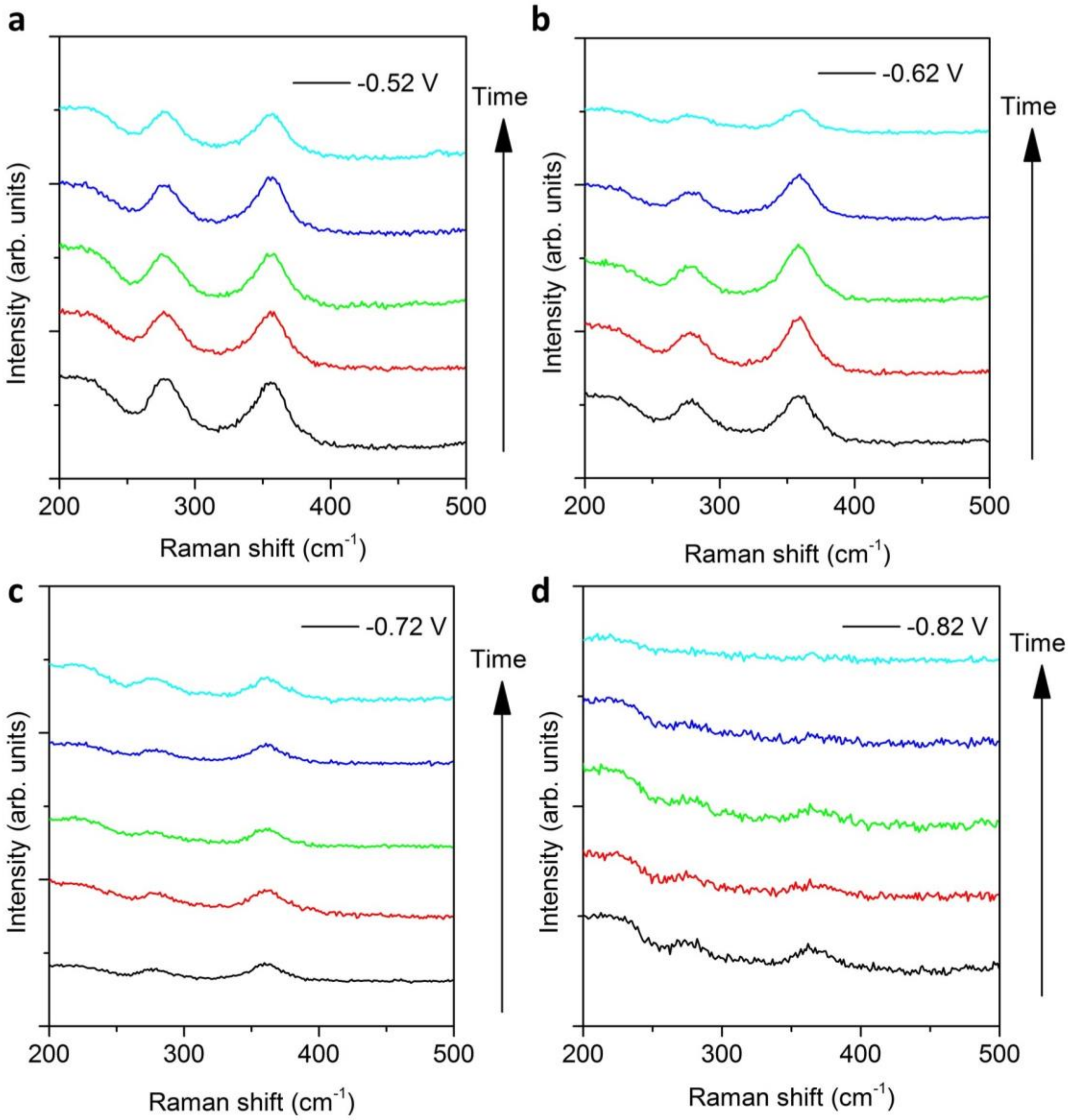

Figure S23. Time-dependent *in situ* surface-enhanced Raman spectra of CO adsorption during CORR at –0.52 V_{RHE} (a), –0.62 V_{RHE} (b), –0.72 V_{RHE} (c) and –0.82 V_{RHE} (d) in a CO-saturated 0.1 M $KHCO_3$ solution. The acquisition time was 20 s. The spectra were recorded every 120 s.

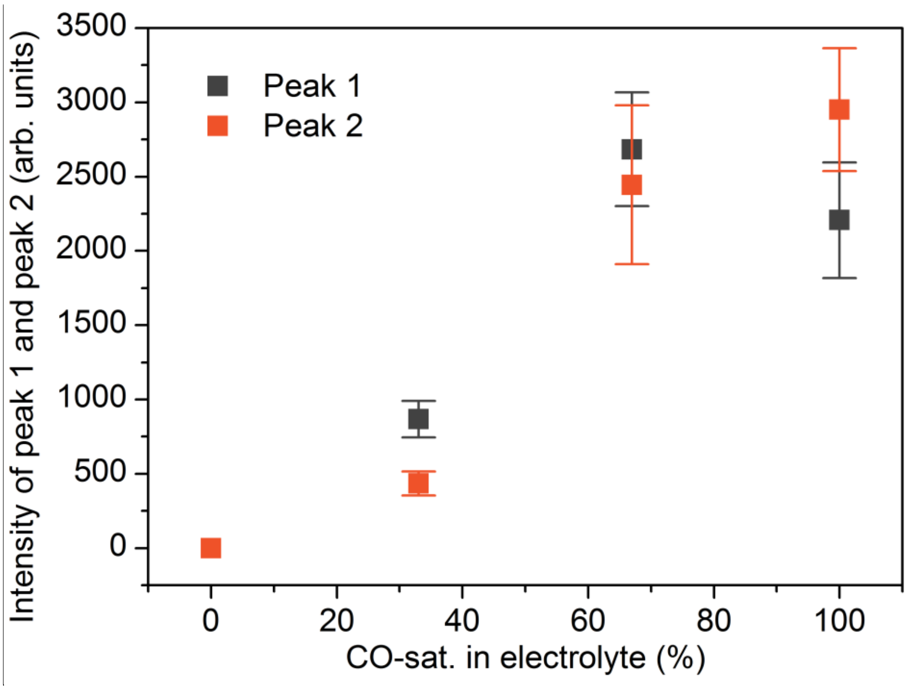

Figure S24. Raman intensity of Peak 2 and Peak 1 in a CO-rich 0.1 M $KHCO_3$ as a function of the volume fraction of CO-saturated $KHCO_3$ in the electrolyte at –0.52 V_{RHE}. We mixed the CO-saturated 0.1 M $KHCO_3$ with the Ar-saturated 0.1 M $KHCO_3$ to prepare the CO-rich 0.1 M $KHCO_3$.

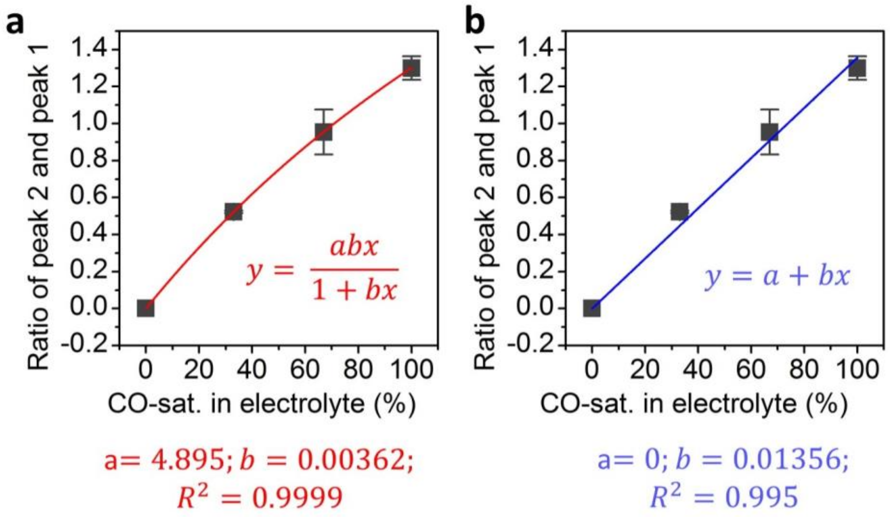

Figure S25. Intensity ratio of the Raman peaks P2 and P1 as a function of the volume fraction of CO-saturated $KHCO_3$ in a CO-rich 0.1 M $KHCO_3$ solution at –0.52 V_{RHE}. We mixed the CO-saturated 0.1 M $KHCO_3$ with the Ar-saturated 0.1 M $KHCO_3$ to prepare the CO-rich 0.1 M $KHCO_3$. Two functions are used for the fitting: Langmuir equation (red) and first order polynomial equation (blue).

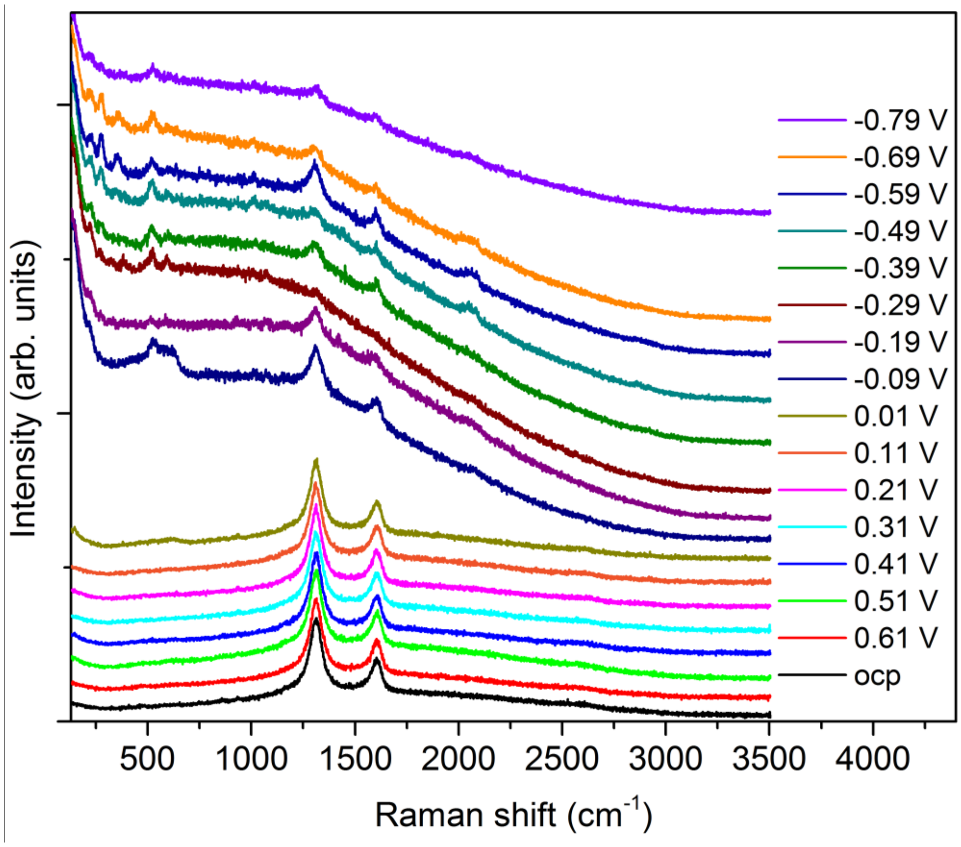

Figure S26. Electrochemical surface-enhanced Raman spectra acquired during CORR over Cu_2O cubes from OCP to –0.79 V_{RHE} in a CO-saturated 0.1 M $NaClO_4$ solution.

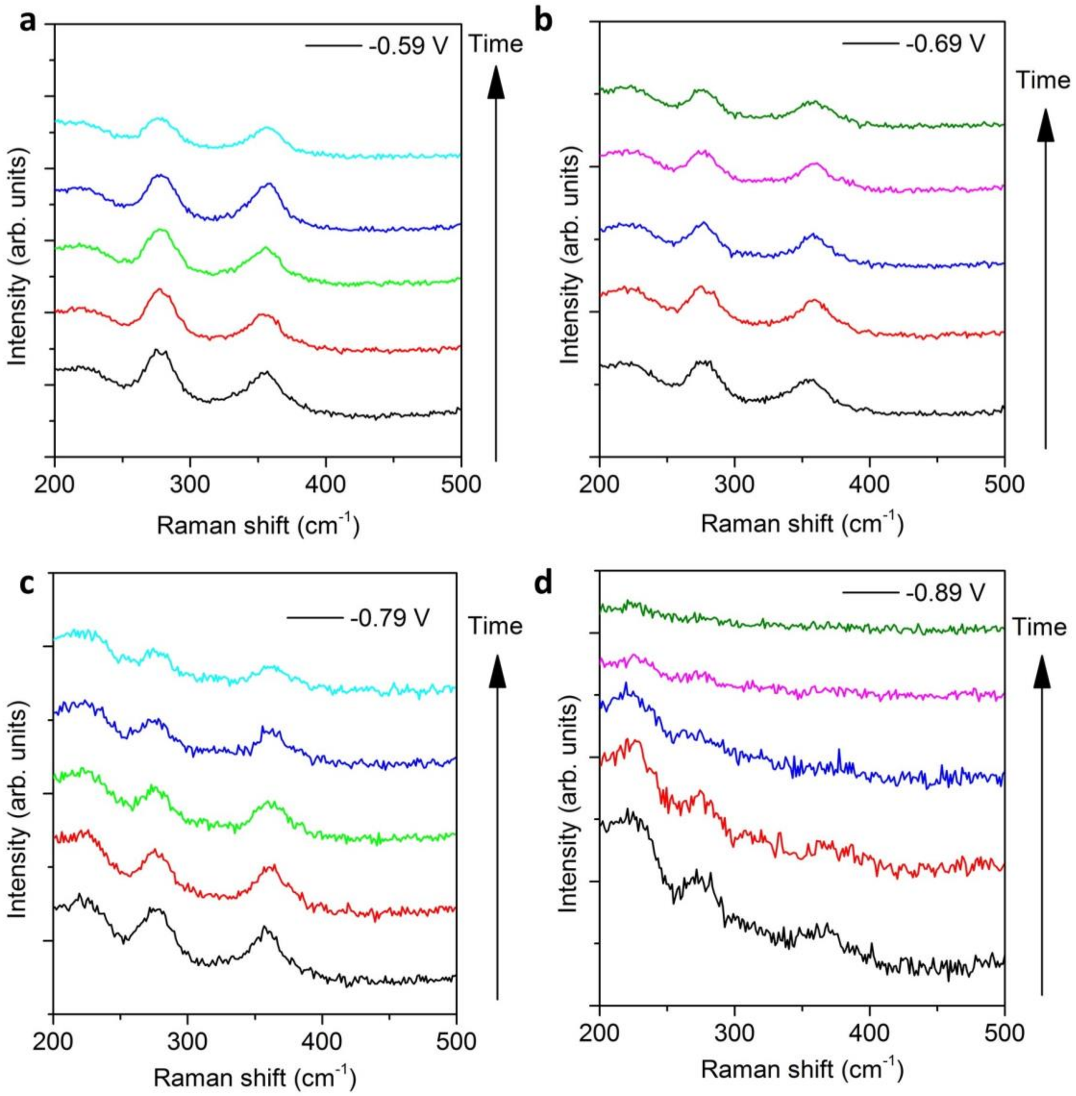

Figure S27. Time-dependent *in situ* Raman of CO adsorption during CORR on Cu_2O cubes at –0.59 V_{RHE} (a), –0.69 V_{RHE} (b), –0.79 V_{RHE} (c) and –0.89 V_{RHE} (d) in a CO-saturated 0.1 M $NaClO_4$ solution. The acquisition time was 20 s. The spectra were recorded every 120 s.

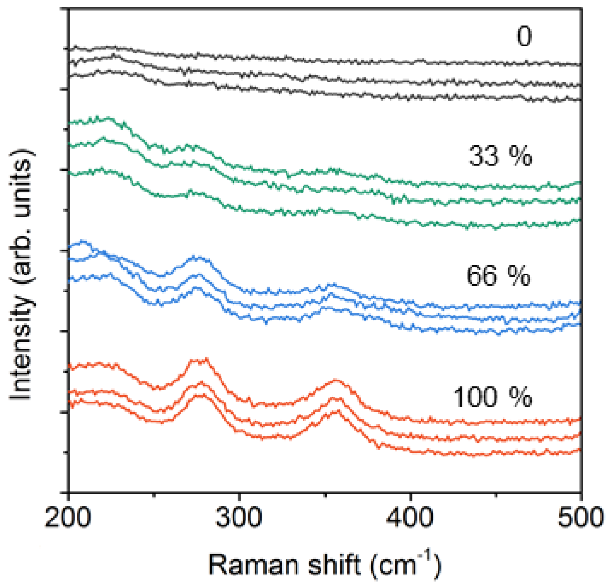

Figure S28. *Operando* Raman spectra of Cu_2O nanocubes in a CO-rich $NaClO_4$ electrolyte with different CO concentrations acquired at –0.59 V_{RHE}. We mixed the CO-saturated 0.1 M $NaClO_4$ with the Ar-saturated 0.1 M $NaClO_4$ to prepare the CO-rich 0.1 M $NaClO_4$ with different CO concentrations. The percentage represents the volume fraction of CO-saturated 0.1 M $NaClO_4$ in the electrolyte from 0 % to 100 %.

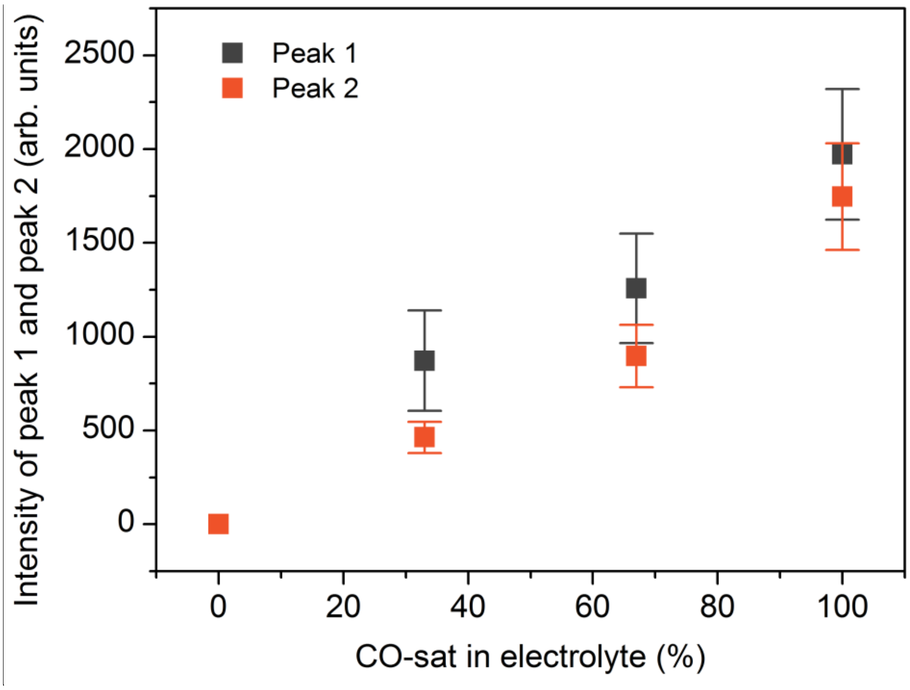

Figure S29. Raman intensity of Peak 2 and Peak 1 as a function of the volume fraction of the CO-saturated $NaClO_4$ in a CO-rich $NaClO_4$ solution at –0.59 V_{RHE}. We mixed the CO-saturated 0.1 M $NaClO_4$ with the Ar-saturated 0.1 M $NaClO_4$ to prepare the CO-rich 0.1 M $KHCO_3$.

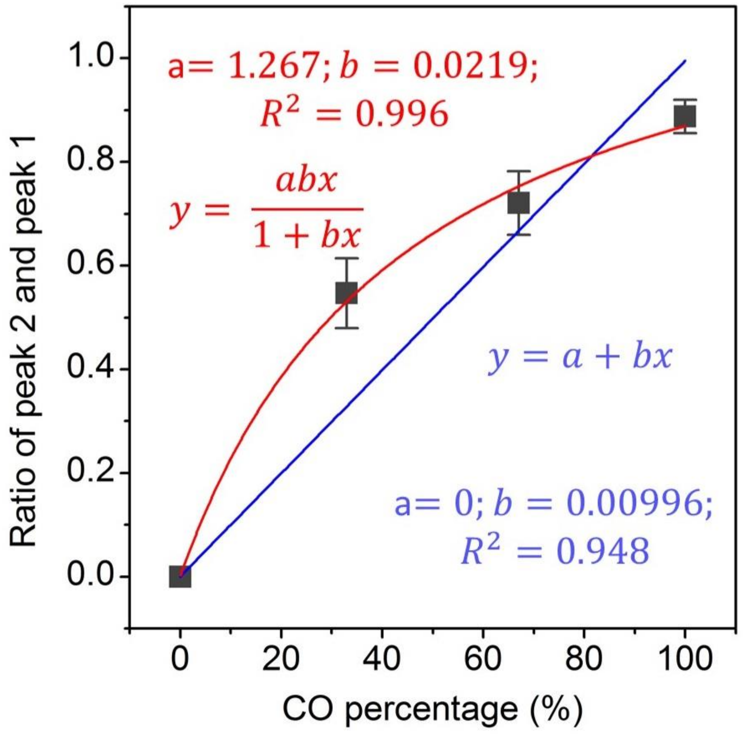

Figure S30. The intensity ratio of the Raman peaks P2 and P1 as a function of the volume fraction of the CO-saturated $NaClO_4$ in a CO-rich $NaClO_4$ solution at –0.59 V_{RHE}. Two functions are used for the fitting: Langmuir equation (red) and first order polynomial equation (blue).

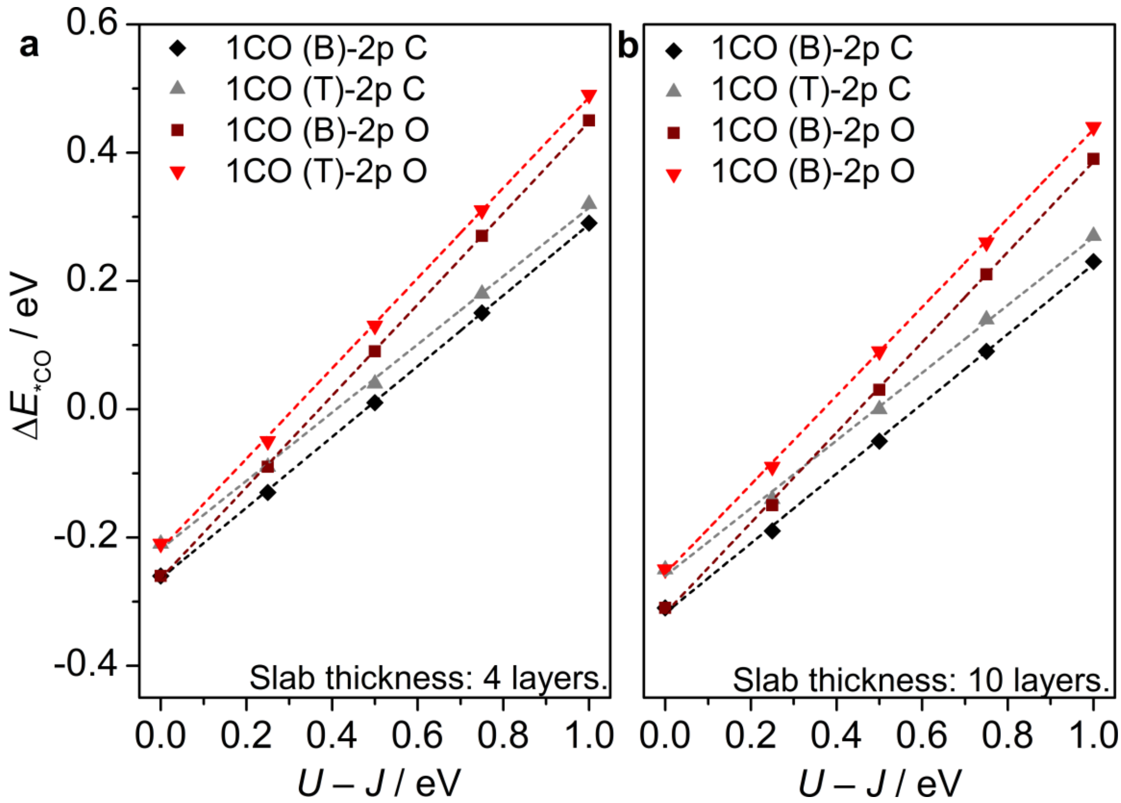

Figure S31. CO binding energy versus Hubbard[14] correction on 2p orbitals of carbon (C) or oxygen (O) atoms in CO at CO surface coverage 0.25 ML for (a) 4 layer and (b) 10-layer Cu slab thickness. A thorough theoretical assessment of site specific CO adsorption through DFT is partially hindered by the underestimation of the HOMO-LUMO gap for CO, which leads to an overestimation of metal/adsorbate interactions.[15] In the literature, a Hubbard correction U_{eff} ($U - J$) for the C or O 2p levels between 0.25 and 1.0 eV has been employed to improve DFT accuracy, leading to the reversal of the adsorption site preference between bridge (no U) and atop (U_{eff} > 0.25) for 0.25 ML CO coverage on Cu(100).[15] We repeated the same benchmark here and for any value of U_{eff} CO adsorption on a bridge site was more favorable than atop.

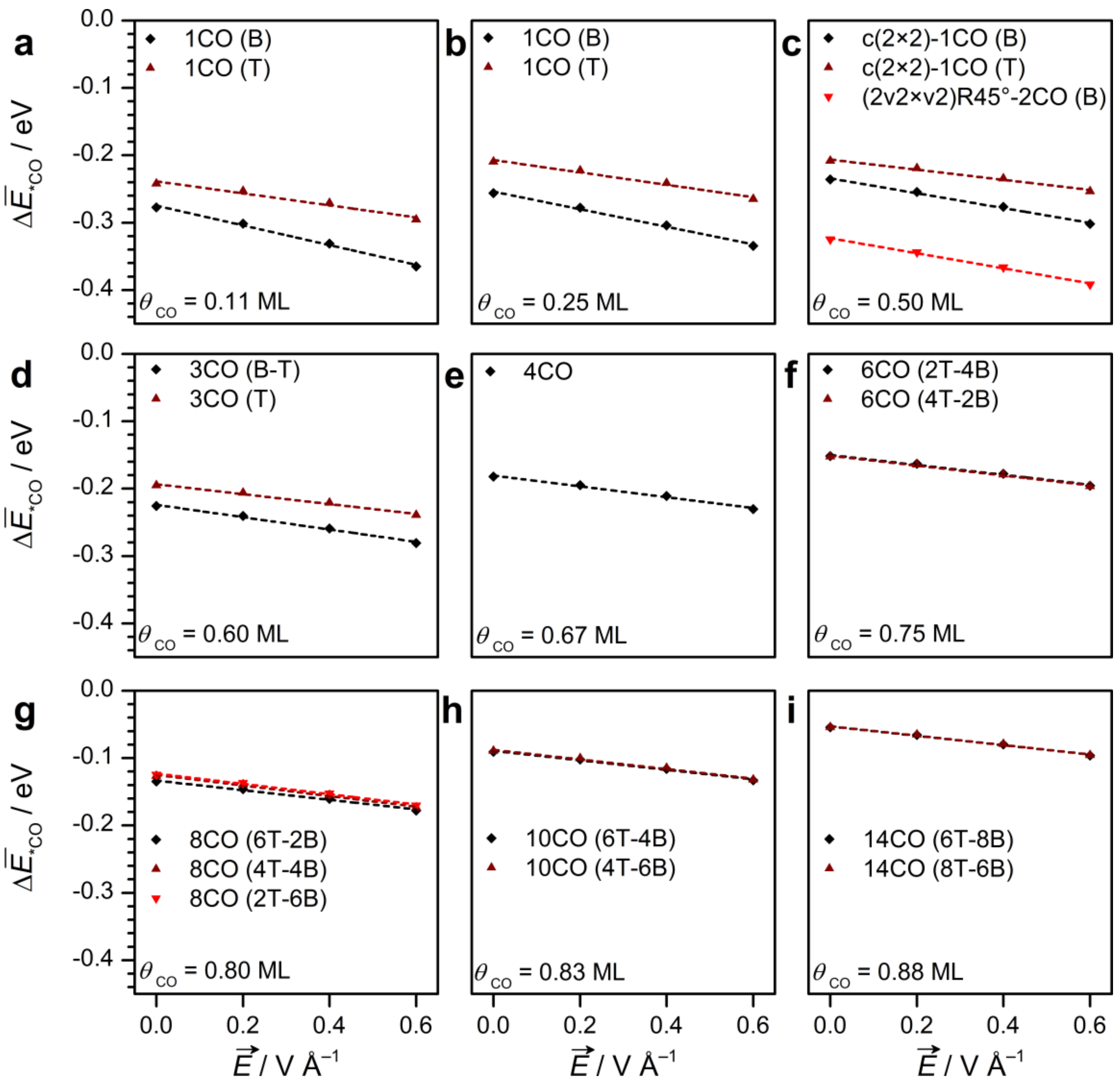

Figure S32. Electric field within the electrical double layer stabilizes CO adsorption on a bridge site *via* electric dipole / field interaction. (a)-(d) For a CO surface coverage lower than 0.67 ML, DFT predicts that CO adsorption is favored on bridge rather than atop sites, in disagreement with experimental results by thermal desorption spectroscopy (TDS) under ultra-high vacuum (UHV) conditions.[15,27] However, CO_{bridge} adsorption is further stabilized by the electric fields applied, which may revert the preferential adsorption site at low CO coverage under electrochemical conditions. Electric field stabilization is due to the higher normal electric dipole moment for CO_{bridge} (0.11 $|e^{-}|$ Å vs 0.03 $|e^{-}|$ Å for CO_{top}).[28]. (e)-(i) At high coverage local CO-CO repulsion lowers CO binding energy and the stabilization effect of the electric field becomes independent of CO adsorption configuration. Regression parameters are reported in Table S3.

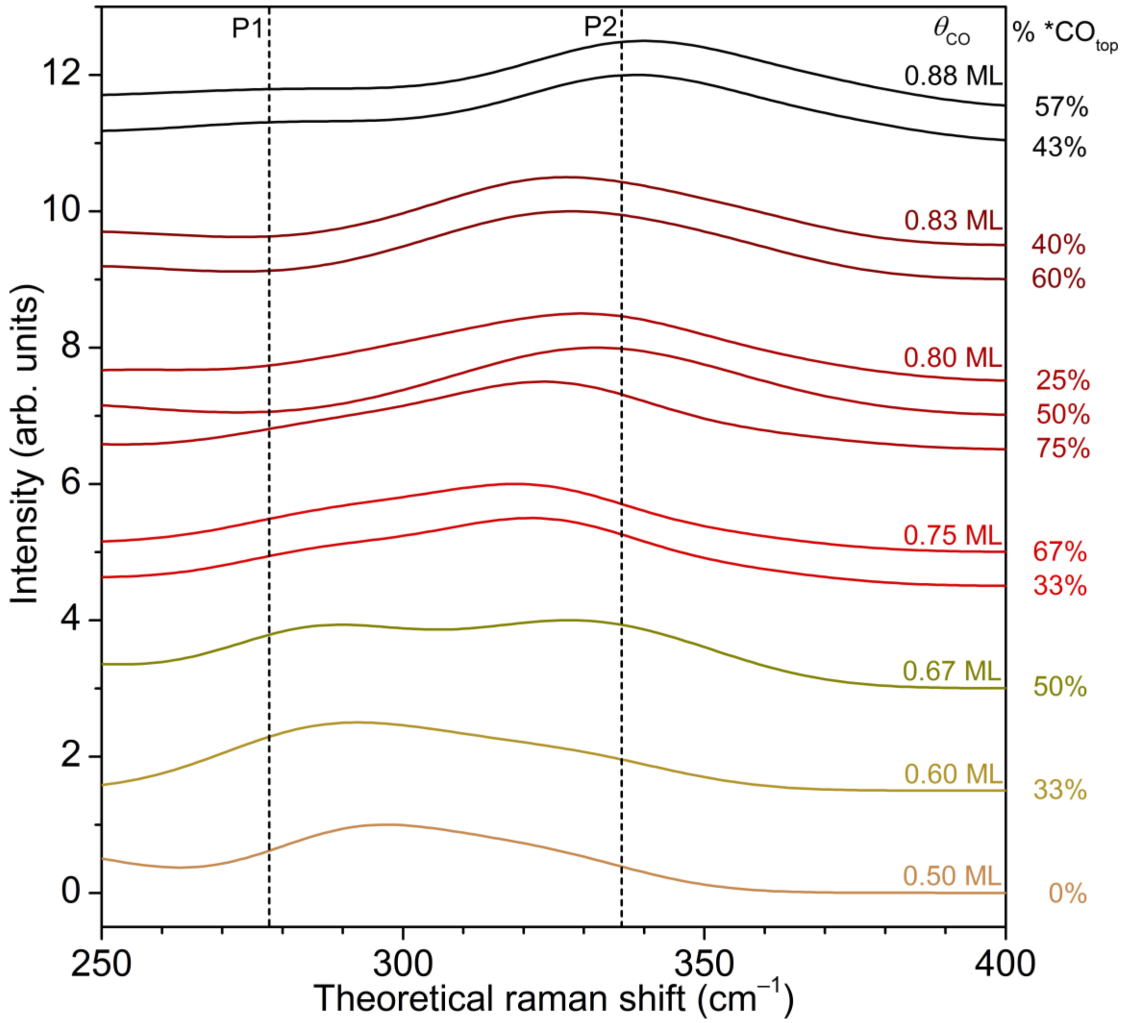

Figure S33. Theoretical vibrational spectra for different CO coverages, θ_{CO}, on Cu(100). A Gaussian function was centered in the Cu-CO stretching band (P2) and CO restricted rotation (P1) vibrational modes and the resulting Gaussian peaks from different adsorption configurations (atop, bridge, etc.) added up to define the spectral lines. For CO surface coverage higher than 0.60 ML, the most stable configuration is a mix of CO_{atop}-CO_{bridge} population, whose adsorption is independent of applied electric field and CO_{atop}/CO_{bridge} ratio (Figure S32, Table S2). Thus, multiple spectra lines are represented. *CO_{atop} represents the percentage of CO adsorbed on an atop Cu site.

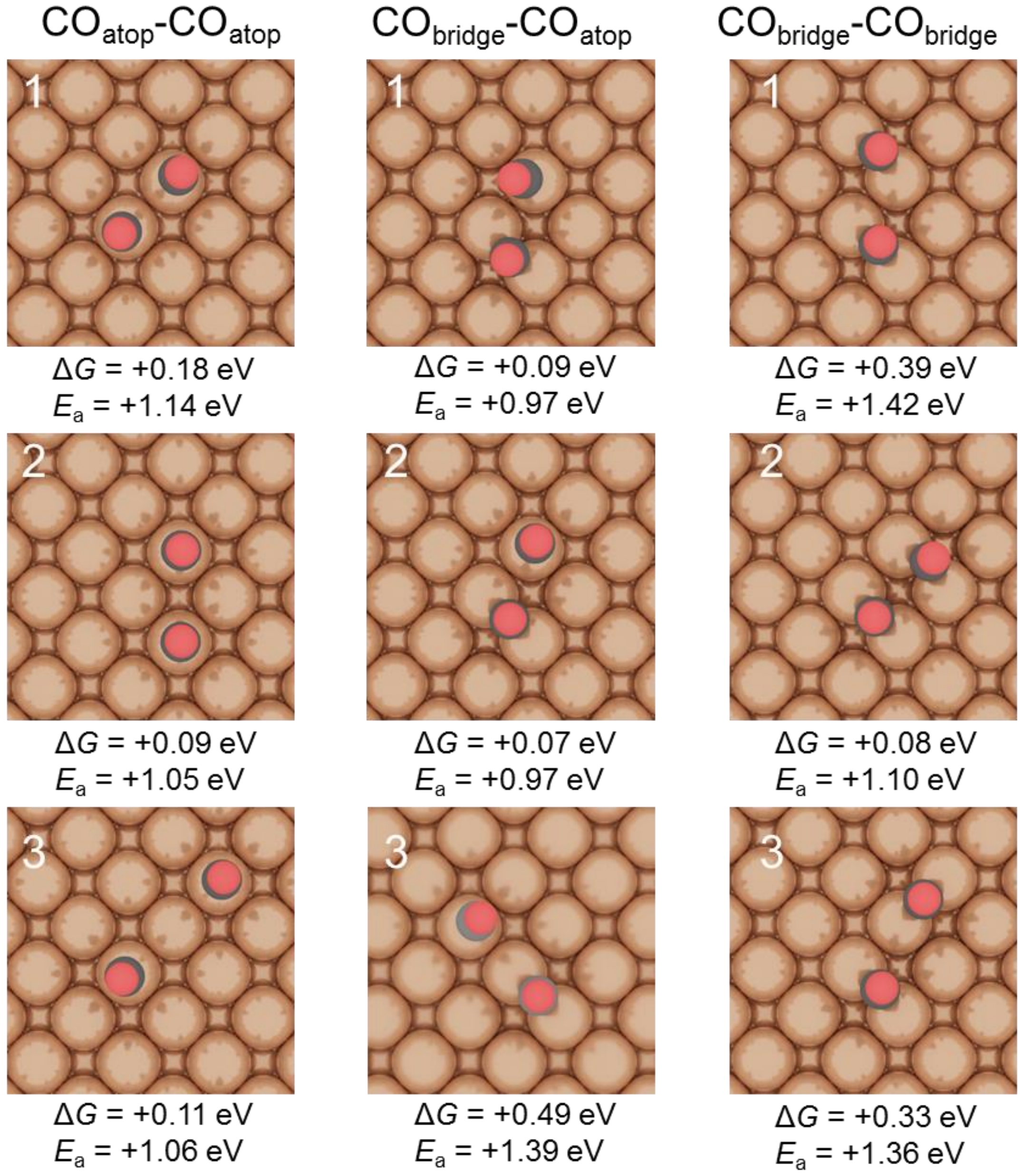

Figure S34. C-C coupling configurations at a surface coverage θ_{CO} of 0.11 ML depending on the CO adsorption site: CO$_{atop}$-CO$_{atop}$ precursor (left); CO$_{bridge}$-CO$_{atop}$ precursor (center); CO$_{bridge}$-CO$_{bridge}$ (right). Each panel is further labeled with the Gibbs free energy of the CO-CO transition state and the activation barrier E_a associated with *OCCO$^-$ formation. High CO surface coverage and electric field effects lower further the activation barriers by 0.2-1.0 eV to ease the process at room temperature.[20-22] Cu atoms are displayed in brown, C in black and O in red.

Tables:

Table S1. DFT vibrational frequencies, v (cm^{-1}), in the region between 200 cm^{-1} and 4000 cm^{-1} for relevant adsorbates on Cu(100). For each adsorbate, we report the surface coverage, θ (ML), and the adsorption configuration.

Adsorbate	θ (ML)	v (cm^{-1})
CO_3^{2-} (bidentate)	0.06	1713, 976, 902, 776, 652, 598, 284, 271, 246, 208
CO_3^{2-} (bidentate)	0.50	1431, 1332, 1022, 749, 461, 413
CO_3^{2-} (monodentate)	0.06	1561, 1230, 835, 735, 574, 433, 216
HCO_3^- (bidentate)	0.06	3609, 1556, 1348, 1188, 1001, 764, 644, 594, 561, 230, 204
HCO_3^- (monodentate)	0.06	3702, 1676, 1294, 1151, 949, 739, 606, 553, 540, 205
H (hollow)	0.06	658, 544, 537
H (hollow)	1.00	637, 451, 444
H (bridge)	0.06	1224, 1183
H (top)	0.06	1757
H (top)	1.00	1763
H_2O (hollow)	0.06	3657, 3475, 1552, 400, 342, 285
H_2O (bridge)	0.06	3690, 3583, 1534
H_2O (top)	0.06	3725, 3617, 1553, 456, 440, 201
OH (hollow)	0.06	3662, 629, 624, 313
OH (bridge)	0.06	3768, 659, 393, 336
CO (bridge)	0.11	1890, 284, 276
CO (top)	0.11	2016, 337, 276, 265
CO (bridge)	0.25	1889, 288, 279
CO (top)	0.25	2010, 335, 278, 258
CO (bridge)	0.50	1888, 319, 283
CO (top)	0.50	2005, 333, 281, 260

Table S2. CO adsorption configurations on Cu(100) for different surface coverages θ_{CO}. The initial configurations were retrieved from a previous study on Pt(100).[18] The systems are defined as a surface supercell, number of adsorbed CO, and the adsorption sites (T: top, B: bridge). $*CO_{atop}$ represents the percentage of CO adsorbed on top Cu sites, vs. CO adsorbed on bridge sites. $\Delta\bar{G}_{*CO}$ (eV) is the average CO binding energy calculated with CO_2, H_2, and H_2O as reference energies. The lowest energy configurations for each surface coverage are highlighted in grey, although at low CO coverage DFT has shown limitations in correctly predicting the preferential adsorption site.[15] For high coverage, different configurations of mixed CO_{atop}-CO_{bridge} populations are isoenergetic within DFT-D2 typical error bars (±0.1 eV). The vibrational spectra associated with Cu-CO stretching band and CO restricted rotation modes are shown in Figure S33.

System	θ_{CO} (ML)	$*CO_{atop}$ (%)	$\Delta\bar{G}_{*CO}$ (eV)
p(3 × 3)-1CO (B)	0.11	0	–0.45
p(3 × 3)-1CO (T)	0.11	100	–0.37
p(2 × 2)-1CO (B)	0.25	0	–0.44
p(2 × 2)-1CO (T)	0.25	100	–0.33
(2√2 × √2)*R*45°-2CO (B)	0.50	0	–0.50
c(2 × 2)-1CO (B)	0.50	0	–0.41
c(2 × 2)-1CO (T)	0.50	100	–0.34
c(5√2 × √2)*R*45°-3CO (1T-2B)	0.60	33	–0.38
c(5√2 × √2)*R*45°-3CO (T)	0.60	100	–0.33
(3√2 × √2)*R*45°-4CO (2T-2B)	0.67	50	–0.32
(4 × 2)-6CO (4T-2B)	0.75	67	–0.28
(4 × 2)-6CO (2T-4B)	0.75	33	–0.28
(5 × 2)-8CO (6T-2B)	0.80	75	–0.26
(5 × 2)-8CO (4T-4B)	0.80	50	–0.26
(5 × 2)-8CO (2T-6B)	0.80	25	–0.25
(6 × 2)-10CO (6T-4B)	0.83	60	–0.21
(6 × 2)-10CO (4T-6B)	0.83	40	–0.21
(8 × 2)-14CO (6T-8B)	0.88	43	–0.17
(8 × 2)-14CO (8T-6B)	0.88	57	–0.17

Table S3. Regression parameters ($\Delta E_{*CO} = a + b \cdot \vec{E}$) for electric field stabilization of CO binding energies at different CO surface coverages and CO adsorption configurations (Figure S32).

System	a (eV)	b (|e⁻| Å)	r^2	χ^2 (10^{-5})
p(3 × 3)-1CO (B)	–0.275 ± 0.003	–0.146 ± 0.008	0.99	2.32
p(3 × 3)-1CO (T)	–0.239 ± 0.004	–0.089 ± 0.011	0.96	4.49
p(2 × 2)-1CO (B)	–0.254 ± 0.003	–0.130 ± 0.007	0.99	2.10
p(2 × 2)-1CO (T)	–0.207 ± 0.003	–0.092 ± 0.008	0.97	2.88
(2√2 × √2)*R*45°-2CO (B)	–0.234 ± 0.002	–0.110 ± 0.005	0.99	1.16
c(2 × 2)-1CO (B)	–0.206 ± 0.002	–0.075 ± 0.006	0.98	1.68
c(2 × 2)-1CO (T)	–0.323 ± 0.002	–0.112 ± 0.005	0.99	1.06
c(5√2 × √2)*R*45°-3CO (1T-2B)	–0.224 ± 0.002	–0.092 ± 0.005	0.99	1.07
c(5√2 × √2)*R*45°-3CO (T)	–0.193 ± 0.002	–0.074 ± 0.006	0.98	1.30
(3√2 × √2)*R*45°-4CO (2T-2B)	–0.181 ± 0.002	–0.080 ± 0.005	0.99	1.08
(4 × 2)-6CO (4T-2B)	–0.150 ± 0.002	–0.073 ± 0.005	0.99	0.88
(4 × 2)-6CO (2T-4B)	–0.151 ± 0.002	–0.073 ± 0.005	0.99	0.89
(5 × 2)-8CO (6T-2B)	–0.133 ± 0.002	–0.072 ± 0.005	0.99	0.94
(5 × 2)-8CO (4T-4B)	–0.125 ± 0.002	–0.079 ± 0.005	0.99	0.87
(5 × 2)-8CO (2T-6B)	–0.123 ± 0.002	–0.077 ± 0.005	0.99	0.86
(6 × 2)-10CO (6T-4B)	–0.089 ± 0.002	–0.070 ± 0.004	0.99	0.72
(6 × 2)-10CO (4T-6B)	–0.088 ± 0.002	–0.071 ± 0.005	0.99	0.91
(8 × 2)-14CO (6T-8B)	–0.053 ± 0.002	–0.070 ± 0.004	0.99	0.73
(8 × 2)-14CO (8T-6B)	–0.053 ± 0.002	–0.070 ± 0.004	0.99	0.68

Table S4. Vibrational frequencies, v, and intensities of the C=O rotation band (P1) for different CO adsorption configurations at high CO coverage. N.D.: Not Defined.

System	θ_{CO} (ML)	v_{P1} (cm^{-1})	Intensity$_{P1}$ (arb. units)
(2√2 × √2)*R*45°-2CO (B)	0.50	298.3 ± 0.3	0.999 ± 0.002
c(5√2 × √2)*R*45°-3CO (1T-2B)	0.60	287.98 ± 0.12	0.981 ± 0.009
(3√2 × √2)*R*45°-4CO (2T-2B)	0.67	289.4 ± 0.7	0.9330 ± 0.0014
(4 × 2)-6CO (4T-2B)	0.75	277.9 ± 1.0	0.44 ± 0.02
(4 × 2)-6CO (2T-4B)	0.75	275 ± 1	0.44 ± 0.04
(5 × 2)-8CO (6T-2B)	0.80	277 ± 1	0.30 ± 0.02
(5 × 2)-8CO (4T-4B)	0.80	N.D.	N.D.
(5 × 2)-8CO (2T-6B)	0.80	294 ± 5	0.47 ± 0.08
(6 × 2)-10CO (6T-4B)	0.83	N.D.	N.D.
(6 × 2)-10CO (4T-6B)	0.83	N.D.	N.D.
(8 × 2)-14CO (6T-8B)	0.88	273.3 ± 0.4	0.288 ± 0.004
(8 × 2)-14CO (8T-6B)	0.88	271.1 ± 0.4	0.276 ± 0.003

Table S5. Vibrational frequencies, v, and intensities of the Cu-CO stretching band (P2) for different CO adsorption configurations at high CO coverage.

System	θ_{CO} (ML)	v_{P2} (cm^{-1})	Intensity$_{P2}$ (arb. units)
(2√2 × √2)*R*45°-2CO (B)	0.50	331.6 ± 0.4	0.49 ± 0.02
c(5√2 × √2)*R*45°-3CO (1T-2B)	0.60	323.5 ± 0.2	0.670 ± 0.013
(3√2 × √2)*R*45°-4CO (2T-2B)	0.67	335.3 ± 0.5	0.951 ± 0.013
(4 × 2)-6CO (4T-2B)	0.75	320.6 ± 0.4	0.9992 ± 0.0007
(4 × 2)-6CO (2T-4B)	0.75	317.6 ± 0.6	0.9991 ± 0.0012
(5 × 2)-8CO (6T-2B)	0.80	322.3 ± 0.3	0.999 ± 0.003
(5 × 2)-8CO (4T-4B)	0.80	332.62 ± 0.03	0.9994 ± 0.0006
(5 × 2)-8CO (2T-6B)	0.80	330.4 ± 0.2	0.9992 ± 0.0008
(6 × 2)-10CO (6T-4B)	0.83	328.58 ± 0.14	0.998 ± 0.002
(6 × 2)-10CO (4T-6B)	0.83	327.6 ± 0.2	0.999 ± 0.002
(8 × 2)-14CO (6T-8B)	0.88	339.83 ± 0.13	0.997 ± 0.003
(8 × 2)-14CO (8T-6B)	0.88	341.55 ± 0.13	0.995 ± 0.004

Table S6. Ratio of P2/P1 for different CO surface coverages, calculated from the intensities reported in Table S4-S5. Uncertainty has been propagated from the standard deviations in Table S4-S5. P2: Cu-CO stretching; P1: C=O rotation. N.D.: Not Defined.

θ_{CO} (ML)	P2/P1 (–)
0.50	0.49 ± 0.02
0.60	0.68 ± 0.02
0.67	1.02 ± 0.02
0.75	2.27 ± 0.02
0.80	2.76 ± 0.62
0.83	N.D.
0.88	3.53 ± 0.08

Table S7. Structural parameters and Bader charges for the initial state CO(1)-CO(2) configuration toward C-C coupling from different CO-CO precursors, shown in Figure S34. CO_b: CO on bridge site. CO_t: CO on atop site.

Precursor	$d_{C\text{-}C}$ (Å)	$d_{C\text{-}O(1)}$ (Å)	$d_{C\text{-}O(2)}$ (Å)	$q_{CO(1)\text{-}CO(2)}$ (|e⁻|)
CO_b-CO_b (n-1)	3.72	1.17	1.17	–0.72
CO_b-CO_b (n-2)	3.09	1.18	1.17	–0.68
CO_b-CO_b (n-3)	3.96	1.17	1.17	–0.72
CO_b-CO_t (n-1)	3.18	1.16	1.17	–0.59
CO_b-CO_t (n-2)	3.19	1.16	1.17	–0.56
CO_b-CO_t (n-3)	3.84	1.16	1.17	–0.58
CO_t-CO_t (n-1)	3.15	1.16	1.16	–0.43
CO_t-CO_t (n-2)	3.61	1.16	1.16	–0.39
CO_t-CO_t (n-3)	5.43	1.16	1.16	–0.40

Table S8. Structural parameters and Bader charges for the transition state CO(1)-CO(2) configuration toward C-C coupling from different CO-CO precursors and the final state $*OCCO^-$. Activation barriers at 0.11 ML CO coverage are reported as well. CO_b: CO on bridge site. CO_t: CO on atop site.

Precursor	$d_{C\text{-}C}$ (Å)	$d_{C\text{-}O(1)}$ (Å)	$d_{C\text{-}O(2)}$ (Å)	q_{OCCO} (|e⁻|)	E_a (eV)
CO_{bridge}-CO_{bridge} (n-1)	1.83	1.18	1.22	–0.75	+1.42
CO_{bridge}-CO_{bridge} (n-2)	1.52	1.20	1.26	–0.87	+1.10
CO_{bridge}-CO_{bridge} (n-3)	1.85	1.18	1.22	–0.73	+1.36
CO_{bridge}-CO_{atop} (n-1)	1.55	1.20	1.24	–0.84	+0.97
CO_{bridge}-CO_{atop} (n-2)	1.53	1.20	1.25	–0.86	+0.97
CO_{bridge}-CO_{atop} (n-3)	1.81	1.18	1.22	–0.80	+1.39
CO_{atop}-CO_{atop} (n-1)	1.69	1.19	1.22	–0.76	+1.14
CO_{atop}-CO_{atop} (n-2)	1.49	1.20	1.26	–0.89	+1.05
CO_{atop}-CO_{atop} (n-3)	1.56	1.20	1.25	–0.84	+1.06
$OCCO^-$	1.34	1.19	1.39	–1.02	–

References

(1) Ke, W. H.; Hsia, C. F.; Chen, Y. J.; Huang, M. H. Synthesis of Ultrasmall Cu_2O Nanocubes and Octahedra with Tunable Sizes for Facet-Dependent Optical Property Examination. *Small* **2016**, *12*, 3530-3534.

(2) Velasco-Vélez, J.-J.; Chuang, C.-H.; Gao, D.; Zhu, Q.; Ivanov, D.; Jeon, H. S.; Arrigo, R.; Mom, R. V.; Stotz, E.; Wu, H.-L.; Jones, T. E.; Roldan Cuenya, B.; Knop-Gericke, A.; Schlögl, R. On the Activity/Selectivity and Phase Stability of Thermally Grown Copper Oxides during the Electrocatalytic Reduction of CO_2. *ACS Catal.* **2020**, *10*, 11510-11518.

(3) Perdew, J. P.; Burke, K.; Ernzerhof, M. Generalized Gradient Approximation Made Simple. *Phys. Rev. Lett.* **1996**, *77*, 3865-3868.

(4) Kresse, G.; Furthmüller, J. Efficiency of Ab-Initio Total Energy Calculations for Metals and Semiconductors Using a Plane-Wave Basis Set. *Comput. Mater. Sci.* **1996**, *6*, 15-50.

(5) Kresse, G.; Furthmüller, J. Efficient Iterative Schemes for Ab Initio Total-Energy Calculations Using a Plane-Wave Basis Set. *Phys. Rev. B* **1996**, *54*, 11169-11186.

(6) Grimme, S. Semiempirical GGA-Type Density Functional Constructed with a Long-Range Dispersion Correction. *J. Comput. Chem.* **2006**, *27*, 1787-1799.

(7) Bucko, T.; Hafner, J.; Lebègue, S.; Ángyán, J. G. Improved Description of the Structure of Molecular and Layered Crystals: Ab Initio DFT Calculations with van der Waals Corrections. *J. Phys. Chem. A* **2010**, *114*, 11814-11824.

(8) Almora-Barrios, N.; Carchini, G.; Błoński, P.; López, N. Costless Derivation of Dispersion Coefficients for Metal Surfaces. *J. Chem. Theory Comput.* **2014**, *10*, 5002-5009.

(9) Garcia-Ratés, M.; López, N. Multigrid-Based Methodology for Implicit Solvation Models in Periodic DFT. *J. Chem. Theory Comput.* **2016**, *12*, 1331-1341.

(10) Garcia-Ratés, M.; García-Muelas, R.; López, N. Solvation Effects on Methanol Decomposition on Pd(111), Pt(111), and Ru(0001). *J. Phys. Chem. C* **2017**, *121*, 13803-13809.

(11) Blöchl, P. E. Projector Augmented-Wave Method. *Phys. Rev. B* **1994**, *50*, 17953-17953.

(12) Kresse, G.; Joubert, D. From Ultrasoft Pseudopotentials to the Projector Augmented-Wave Method. *Phys. Rev. B* **1999**, *59*, 1758-1775.

(13) Monkhorst, H. J.; Pack, J. D. Special Points for Brillouin-Zone Integrations. *Phys. Rev. B* **1976**, *13*, 5188-5192.

(14) Dudarev, S.; Botton, G.; Savrasov, S.; Humphreys, C.; Sutton, A. Electron-Energy-Loss Spectra and the Structural Stability of Nickel Oxide: An LSDA+ U Study. *Phys. Rev. B* **1998**, *57*, 1505.

(15) Gajdoš, M.; Hafner, J. CO Adsorption on Cu(111) and Cu(001) Surfaces: Improving Site Preference in DFT Calculations. *Surf. Sci.* **2005**, *590*, 117-126.

(16) Feibelman, P. J. Surface-Diffusion Mechanism versus Electric Field: Pt/Pt(001). *Phys. Rev. B* **2001**, *64*, 125403.

(17) Hori, Y.; Murata, A.; Yoshinami, Y. Adsorption of CO, Intermediately Formed in

Electrochemical Reduction of CO_2, at a Copper Electrode. *J. Chem. Soc., Faraday Trans.* **1991**, *87*, 125-128.
(18) Sumaria, V.; Nguyen, L.; Tao, F. F.; Sautet, P. Optimal Packing of CO at a High Coverage on Pt(100) and Pt(111) Surfaces. *ACS Catal.* **2020**, *10*, 9533-9544.
(19) Makov, G.; Payne, M. Periodic Boundary Conditions in Ab Initio Calculations. *Phys. Rev. B* **1995**, *51*, 4014-4022.
(20) Jiang, K.; Sandberg, R. B.; Akey, A. J.; Liu, X.; Bell, D. C.; Nørskov, J. K.; Chan, K.; Wang, H. Metal Ion Cycling of Cu Foil for Selective C–C Coupling in Electrochemical CO_2 Reduction. *Nat. Catal.* **2018**, *1*, 111-119.
(21) Resasco, J.; Chen, L. D.; Clark, E.; Tsai, C.; Hahn, C.; Jaramillo, T. F.; Chan, K.; Bell, A. T. Promoter Effects of Alkali Metal Cations on the Electrochemical Reduction of Carbon Dioxide. *J. Am. Chem. Soc.* **2017**, *139*, 11277-11287.
(22) Sandberg, R. B.; Montoya, J. H.; Chan, K.; Nørskov, J. K. CO-CO Coupling on Cu Facets: Coverage, Strain and Field Effects. *Surf. Sci.* **2016**, *654*, 56-62.
(23) Olsen, R.; Kroes, G.; Henkelman, G.; Arnaldsson, A.; Jónsson, H. Comparison of Methods for Finding Saddle Points without Knowledge of the Final States. *J. Chem. Phys.* **2004**, *121*, 9776-9792.
(24) Peterson, A. A.; Abild-Pedersen, F.; Studt, F.; Rossmeisl, J.; Nørskov, J. K. How Copper Catalyzes the Electroreduction of Carbon Dioxide into Hydrocarbon Fuels. *Energy Environ. Sci.* **2010**, *3*, 1311-1315.
(25) Nørskov, J. K.; Rossmeisl, J.; Logadottir, A.; Lindqvist, L.; Kitchin, J. R.; Bligaard, T.; Jónsson, H. Origin of the Overpotential for Oxygen Reduction at a Fuel-Cell Cathode. *J. Phys. Chem. B* **2004**, *108*, 17886-17892.
(26) Cuesta, A.; Dhamelincourt, P.; Laureyns, J.; Martínez-Alonso, A.; Tascón, J. M. D. Raman Microprobe Studies on Carbon Materials. *Carbon* **1994**, *32*, 1523-1532.
(27) Vollmer, S.; Witte, G.; Wöll, C. Determination of Site Specific Adsorption Energies of CO on Copper. *Catal. Lett.* **2001**, *77*, 97-101.
(28) Gunathunge, C. M.; Ovalle, V. J.; Li, Y.; Janik, M. J.; Waegele, M. M. Existence of an Electrochemically Inert CO Population on Cu Electrodes in Alkaline pH. *ACS Catal.* **2018**, *8*, 7507-7516.
